# Physiology and the future of animals in shifting-sand deserts: respiration, energetics and water balance

**DOI:** 10.1093/conphys/coag058

**Published:** 2026-08-20

**Authors:** Duncan Mitchell, Mary K Seely, Shane K Maloney

**Affiliations:** Brain Function Research Group, Department of Physiology, University of the Witwatersrand Medical School, 7 York Road, Parktown 2193, Johannesburg, South Africa; School of Human Sciences, University of Western Australia, 35 Stirling Highway, Crawley 6009, Perth, WA, Australia; 55 Libertina Amathila Avenue, Swakopmund, Namibia; Brain Function Research Group, Department of Physiology, University of the Witwatersrand Medical School, 7 York Road, Parktown 2193, Johannesburg, South Africa; School of Human Sciences, University of Western Australia, 35 Stirling Highway, Crawley 6009, Perth, WA, Australia

**Keywords:** Beetle, cellulose, desertification, detritus, dew, fog, lizard, mole, oxygen, sand-swimming

## Abstract

The many invertebrates and reptiles of the shifting-sand deserts, and their rare mammals, live in and on the sand. Their conservation requires a thorough understanding of the physiological mechanisms that not only enable them to function, but that may buffer them against future change. The almost-spherical shape of the aeolian sand grains of shifting-sand deserts enables gas diffusion from the surface to maintain a normoxic environment deep in the sand, with sufficient volume to sustain sand-swimming animals, even the mammals. In the resource-poor desert landscapes, cellulose is the dominant source of metabolic energy for herbivores, much of it contained in detritus that can originate hundreds of kilometres from the desert. Carnivores and omnivores consume mainly detritivores, with termites often contributing substantially to their diets. Many animals of shifting-sand deserts manage their body water by the acquisition of metabolic and preformed water, balanced against limited loss of body water via integument, excreta and respiration. Those that do drink usually use droplets from fog or dew. Some small invertebrates extract water vapour from unsaturated air. The animals have evolved mechanisms to counteract osmotic shock when they consume pure water that can constitute more than 40% of body mass in a single drink. In a companion review, we discuss how the animals move and how they cope with potentially lethal desert heat and cold in and on the sand. We need to conserve their fortresses in the sand, and, anomalously, it is food, not water, that requires attention.

This article is linked to “Physiology and the future of animals in shifting-sand deserts: living, moving and thermoregulating in the sand” (https://doi.org/10.1093/conphys/coag057).

## Introduction

The animal residents of shifting-sand deserts are active on the surface and swim in the loose sand. They cannot construct unsupported burrows in it. Their sand is a fortress (see Mitchell *et al.,* 2026). It is very difficult for invaders to enter their fortress but equally its residents cannot easily leave it for other habitats. If their fortress is threatened by climate change or by other human abuse, they have nowhere to go. Their survival will depend on whether they have the physiological flexibility to cope with changes in their habitat. An assessment of whether they do have that flexibility requires a thorough understanding of the physiological mechanisms that they use to cope with the habitat in a shifting-sand desert that lacks water, is poorly resourced by food, and sometimes lethally hot or cold. Also, they have to be able to move and breathe in the sand.

In a companion paper (Mitchell *et al.,* 2026), we review the mechanics of how quiescent buriers and true sand swimmers move on and in the sand. We also address the proposition that the usual reason for them to retreat into the sand is because it is either a thermal haven or a hygric haven. Here, we explore how they breathe in sand, sometimes deep below the surface, how they acquire and manage metabolic energy in a habitat that has low productivity, especially when they have to meet the metabolic demand of swimming in a granular substrate, and how they acquire and manage body water in a dry habitat. We conclude with more lessons about the conservation of the animals of shifting-sand deserts that we have learnt from their physiology.

## Breathing in the sand

**Figure 1:**
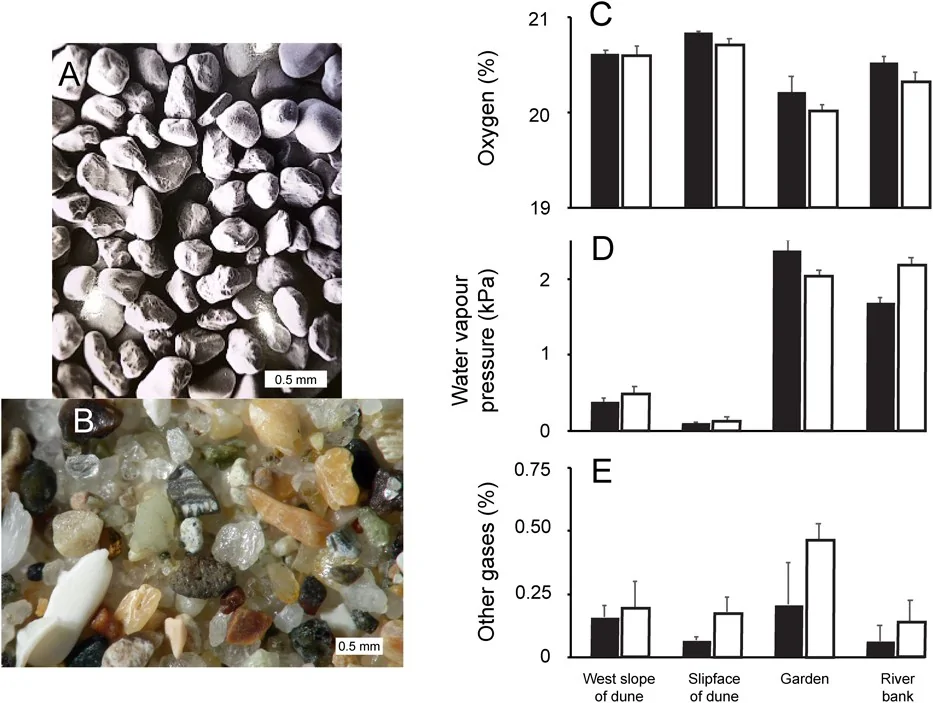
(**A** and **B**) Micrographs of sand granules. (A) Aeolian granules from the slipface of a Namib Desert dune. The ‘approximately spherical’ shape reduces friction and increases air space. (B) For comparison, granules from Pismo Beach, California, USA; jagged not round, less uniform in size and less air space. Photo: Public domain, Author Wilson44691: Mark A. Wilson, Department of Geology, The College of Wooster, https://commons.wikimedia.org/wiki/File:PismoBeachSand.JPG (**C**–**E**) Atmospheric gases at a depth of 100 mm (solid bars) and 300 mm (open bars) below the surface at sites near the Gobabeb Namib Research Institute (−23.6°S, 15.0°E): west (windward) dune slope, dune slip face, garden of Institute laboratory and bank of the dry riverbed of the Kuiseb River. Oxygen partial pressure (C) differed very little from that in free air. Water vapour pressure (D) was very low in dune sand. The ‘other gases’ (E) in the garden soils likely were carbon dioxide and methane produced by microorganisms that are absent from the desert sand.

One would expect any invertebrate to be able to breathe in the topmost layers of shifting-sand deserts because their metabolic demands are low and the sand is porous and close to the surface. The task is made easier for the burying tenebrionid beetles, or at least those of the Namib Desert, because of their extraordinarily low demand for oxygen. Extrapolation of the rates of oxygen consumption measured by Louw *et al.* (1986) for the tenebrionid beetle *Onymacris unguicularis* implies that it consumes just 0.5 ml of oxygen per day. One would not expect vertebrates to be able to breathe underground because their metabolic demands are higher and they would not be surrounded by enough air except in a burrow. Three species of mammal are known to breath while they are swimming deep in loose desert sand, the Namib golden mole *Eremitalpa granti namibensis* in Africa, and two species of marsupial mole *Notoryctes typhlops* and *Notoryctes caurinus* in Australia (see Mitchell *et al.,* 2026). It is only in shifting-sand deserts that mammals can breathe underground. That mammals can breathe underground in shifting-sand deserts results from the rounding of sand granules by aeolian forces, and the subsequent creation of air spaces between the granules (see Fig. 1; Robinson & Seely, 1980; Mitchell *et al.,* 2026). The dunes sagebrush lizard *Sceloporus arenicolus*, which swims in the dunes of New Mexico and adjacent Texas, USA, is confined to areas of coarse sand, with its larger air spaces (Ryberg & Fitzgerald, 2015).

The diffusion of atmospheric air downwards between gaps in sand granules can result in oxygen partial pressures almost equal to that in free air down to at least 800 mm (Seymour, 1973*a*), and not just under loose sand. What differs in the shifting-sand deserts is not so much oxygen partial pressure as oxygen volume. The air space can make up more than 40% of the sand volume (Seymour & Seely, 1996; Maladen *et al.,* 2009). In the arid deserts, there are few active microorganisms that consume oxygen from that space or add carbon dioxide and methane to it (Seymour & Seely, 1996). Thus, provided that an animal can achieve the mechanical forces that are required to ventilate and can prevent its airways being clogged by sand granules, it will have adequate access to air beneath the desert sand. *Uma* lizards, which are quiescent buriers (Mitchell *et al.,* 2026), appear not to require any special respiratory physiology to acquire oxygen in the sand (Pough, 1969). In the laboratory, the rate of oxygen consumption of the sand-swimming Namib Desert lizards *Gerrhosaurus skoogi* at rest was the same when the lizards were buried in the sand as it was when they were on the sand surface (Clarke & Nicolson, 1994). A sealed and occupied burrow constitutes a much more hostile respiratory environment than does the shifting desert sand (Nevo, 1979). For more detail about how the animals overcome the mechanical forces and maintain ventilation, see Appendix 1 of the supplementary material.

It is a surprise to find anurans that are residents of dry shifting-sand habitats, but some are, notably species of the spadefoot toads of the *Scaphiopodidae* (North America) and *Limnodynastidae* (Australia) families. They do not dig burrows but reverse into the sand, until they are covered completely. How do they breathe? Seymour (1973*a*) measured the oxygen concentration adjacent to buried *Scaphiopus* spadefoot toads in their natural habitat in Arizona, USA, and found that the oxygen concentration usually was more than 20%, even at a depth of 800 mm. Right after they buried, *Scaphiopus couchii* and *Scaphiopus hammondii* used the same breathing technique as they used on the surface, namely buccal movements and movements of the flanks, but all movements ceased after a day of burial, with the toads apparently dormant (Seymour, 1973*a*). Once ventilatory movements stopped, the toads continued to exchange oxygen and carbon dioxide through their skin. Seymour (1973*b*) also measured the metabolic rate of *S. hammondii* while they were resting, during forced movement in a rotating drum and during natural burying. The forced movement was used not to mimic their natural movement, but to generate the maximum rate of oxygen consumption; when they bury naturally, the toads dig in intermittent bursts. Their metabolic rates usually were well below those of mammals, but during the bursts they achieved metabolic rates that overlapped with those of mammals, but only of mammals at rest (Seymour, 1973*b*).

Like the *Scaphiopodidae* and *Limnodynastidae,* the *Pelobatidae* do not burrow, but bury in the ground without leaving a cavity. They are a family of spadefoot toads that inhabit the western Palaearctic. To avoid freezing, Pallas’ spadefoot toad *Pelobates vespertinus* overwinters at a depth of up to 2 m. It is extraordinarily resistant to hypoxia, being sluggishly active at an atmospheric oxygen concentration of 3% (Berman *et al.,* 2024). At 2 m underground, however, the oxygen concentration in loamy sand in its native habitat did not drop below 19% (Berman *et al.,* 2024); the selection pressure that led to the evolution of resistance to hypoxia was unlikely to be related to burial. Therefore, it is not just in shifting-sand deserts that the air surrounding a buried animal has an oxygen concentration close to that of the free atmosphere.

How long can sand-swimming vertebrates remain stationary before the local respiratory gases reach a concentration that is incompatible with life? The rate of diffusion of oxygen into the sand, and of carbon dioxide out of it, depends on the partial pressure difference between the sand and the surface and on the molecular weight of the gas. When it was measured, the effect of the sand on the rate of diffusion was the same for both gases (Penman, 1940). Even with the generous air space of loose desert sand, it takes time for surface oxygen to diffuse into the sand because the oxygen concentration difference between the surface and the air in the sand at depth cannot be more than 21% (Vihar *et al.,* 2015). We displaced all of the oxygen at 300 mm below the surface of the slip face of a Namib Desert dune by injecting a bolus of carbon dioxide and measured the time that it took for air to displace that carbon dioxide, a technique like that used by Louw *et al.* (1986) in the laboratory. It took about 40 min for the oxygen partial pressure to recover to 95% of the surface pressure (Goelst K, Mitchell D, Seely MK, unpublished). Rathbun and Rathbun (2007) reported that six radiotagged Namib golden moles, in their natural habitat, did not change position on 43% of days, admittedly in winter months. In the laboratory, at 400 mm below sand from the Namib Desert, the partial pressure of oxygen in the air surrounding Namib golden moles at rest remained stable and just below surface partial pressure for at least 800 min (Seymour & Seely, 1996). Therefore, resting golden moles would not have to seek fresh air. Golden moles do become torpid (Fielden *et al.,* 1990*b*; Seymour & Seely, 1996; Rathbun & Rathbun, 2007). Then, their depressed oxygen demand would allow them to remain immobile for even longer. Their low metabolic rate cannot be an adaptation to hypoxia, as has been suggested (Fielden *et al.,* 1990*b*), because they are not hypoxic, even when they are under the sand (Seymour & Seely, 1996). When they swim in the sand, their oxygen demand will increase, but then they will constantly access ‘fresh air’ when they move. Vihar *et al.* (2015) pointed out that sandfish moved about in the sand and so regularly entered areas of ‘fresh air’. Whether the animals have to move is important to conservationists who may need to track them.

## The management of energy metabolism in and on the sand

In any biome, animals that have low dispersal capabilities do not have access to more metabolic energy than is generated by the primary production of that biome. Deserts contribute only 1–2% of the world’s terrestrial primary productivity despite covering up to a third of the land mass (Crawford & Gosz, 1982). The main determinant of the primary productivity of shifting-sand deserts is the availability of water for plant growth. In shifting-sand deserts, the biomass increases temporarily, but massively, following rainfall events that are sufficient to stimulate explosive plant germination and growth (Seely & Louw, 1980; Hadley & Szarek, 1981; Smith & Morton, 1990; Aushiku *et al.,* 2015). In the Namib Desert, such events may occur only once every 40 years (Mitchell *et al.,* 2020), while near Alice Springs, Australia, they occur at intervals of no more than 5 years (Smith & Morton, 1990). In the Atacama Desert, flowering episodes take place every 5–7 years (Vidal *et al.,* 2011). In the long arid intervals between such events, not only does the biomass decline but it shifts position on sand dunes and between the surface and underground sites (Southgate *et al.,* 1996). Although some of the terrain of shifting-sand deserts is perpetually vegetationless, perennial plants, like some grasses and succulents, persist throughout those arid intervals at some sites (Southgate *et al.,* 1996). Possible reasons for their persistence are that they grow in substrate so poor in nutrients that their tissues are unattractive to herbivores (Smith & Morton, 1990) or that they contain toxic secondary compounds (Meyer & Karasov, 1991).

Over their lifetime, much more of the daily turnover of metabolic energy of most animals is directed to growth and maintenance than it is to movement, even in active animals. ‘Over evolutionary timeframes, variation in daily locomotor activity does not predict variation in daily expenditure’ (Pontzer, 2025, abstract). Over shorter time frames, activity can be the major contributor to metabolic rate. In what probably was the first study of distance-energy budgets (as distinct from time-energy budgets), Seymour *et al.* (1998) showed that resting metabolism accounted for 37% of the daily energy turnover of Namib golden moles, and sand-swimming accounted for 11%, so foraging and other surface activities must have accounted for 52%. Thus, the total activity accounted for 63% of the daily energy turnover. Gila monsters *Heloderma suspectum*, of the Mojave and other deserts of North America, where their preferred habitat is rocky scrub rather than loose sand, devote two-thirds of their daily energy expenditure to surface activity (Gienger *et al.,* 2014).

### Where do the animals of shifting-sand deserts obtain metabolic energy?

That there is any animal life in shifting-sand deserts hinges on one molecule, cellulose. By far, the majority of animals that live in shifting-sand deserts obtain their metabolic energy from that molecule. Carnivores (including insectivores) obtain some or all of their energy by eating animals that are cellulose-dependent. Cellulose is a straight-chain polysaccharide of variable length, which is synthesized by plants as a structural molecule. It is not soluble in water and remarkably indigestible to most animals, especially vertebrates (Watanabe & Tokuda, 2001). To break down cellulose to D-glucose, which provides the metabolic energy to cellulose-dependent animals, three families of cellulase enzymes are needed to work in series. How desert animals achieve that breakdown is described in Appendix 2; even in invertebrates, the enzymes are active mostly in symbionts.

**Figure 2:**
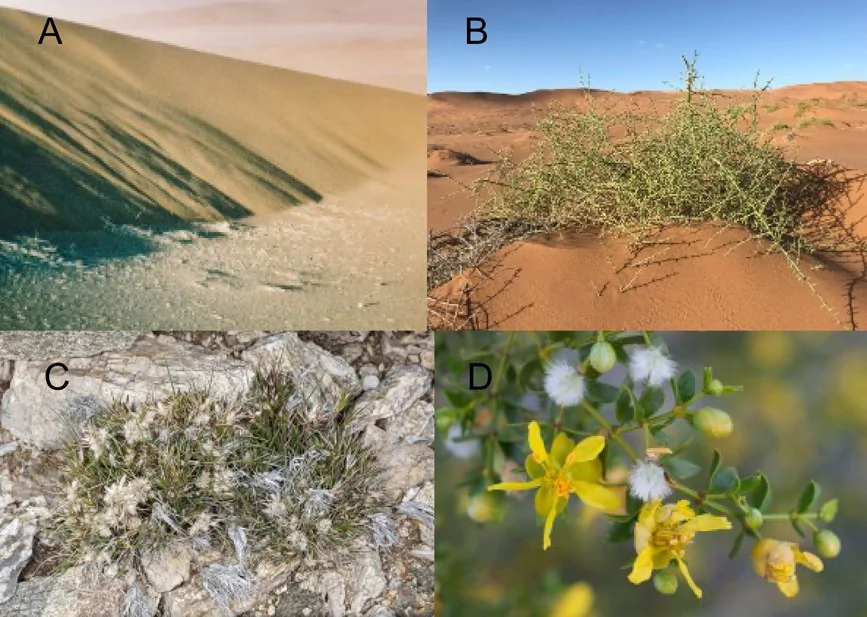
(**A**) Detritus clumps at the base of the slip face of a vegetationless Namib Desert dune, where it was deposited by local wind eddies on the slip face (Seely & Louw, 1980; Southgate *et al.,* 1996). Photo: Duncan Mitchell, copyright retained. (**B**) Nara (*Acanthosicyos horridus*), a leafless cucurbit plant of the Namib Desert, the spines of which are the primary food source for the lizard *G. skoogi* (see Fig. 2). Photo: Carole Roberts, copyright retained. (**C**) Desert fluff-grass *D. pulchella,* from the deserts of western North America, a desert plant that does not thrive in the absence of termites. Photo: Wikimedia Commons (Toughsquid—Own work, CC BY-SA 4.0, https://commons.wikimedia.org/w/index.php?curid=147092266) (**D**) Creosote bush *L. tridentata*, also from the deserts of western North America, the bitter leaves of which are consumed voraciously by termites but are unpalatable to vertebrates. Photo: Wikimedia Commons (Eric in SF—Own work, CC BY-SA 3.0, https://en.wikipedia.org/wiki/Larrea_tridentata#/media/File:Larrea_tridentata_Furnace_Creek.jpg).

Sand dunes may be free of vegetation but are not free of resident animals, so where do those animals find cellulose? On sand dunes, the main source of cellulose is plant material in detritus (Seely & Louw, 1980; Hadley & Szarek, 1981), which consists mainly of decomposing organic material of plant origin but also may contain animal and faecal fragments and sand. Scoured by the wind and ground by the sand, the form of detritus varies from fibrous pads (see Fig. 2) to powder. It can lie on the sand surface in discontinuous mobile patches (Crawford & Gosz, 1982; Crawford, 1988) or be buried, and, indeed, cycle between the surface and the subsurface (Lawrence, 1959; Seely & Louw, 1980). How much cellulose is present on a surface, but not that underground, can be measured by satellite imaging, using an absorption index based on the spectral qualities of cellulose (Noy *et al.,* 2025). It is a reliable source of food in shifting-sand deserts, available even when primary plant production is very low (Seely, 1983). After a long dry period in the Namib Desert, the biomass of detritus on the dunes was estimated to be 4 kg·ha^−1^, rising to 27 kg·ha^−1^ after 100  mm of rain (Seely, 1983).

Although the sources of detritus are poorly known for most deserts (Crawford & Taylor, 1984), much of the detritus of the central Namib sand sea is blown into the desert, from savannah highlands hundreds of kilometres to its east, by intermittent but powerful offshore winds (Louw & Holm, 1972). That the primary food source in a shifting-sand desert may have its origins outside the desert will have consequences for the conservation of the animals in that desert. The Namib Desert is unlikely to be the only desert that has a distant sources of detritus.

For such a ubiquitous and important food source in deserts, little is known about the biochemistry of detritus. It has a protein concentration that is sufficiently low to limit the growth and reproduction of an animal when it is the primary food source (Seely & Louw, 1980). The energy value of samples of Namib detritus can be calculated from numbers in Seely and Louw (1980); it was 16.5 kJ·g^−1^ of dry matter, similar to that of English wheat (McCance & Walsham, 1948). The fact that animals of shifting-sand deserts will eat some detritus even when more-palatable food is available probably implies that it contains nutrients that are absent or deficient in those other foods; sodium is a candidate (Nagy *et al.,* 1991).

Whatever the nutritional strengths and deficiencies of detritus, it nourishes the most numerous of all animals of shifting-sand deserts, the detritivores (Crawford, 1979). In some deserts, the sustainable mass of detritus is determined by how fast it is removed by detritivores (Sagi *et al.,* 2019). While most detritivores are invertebrates, detritus forms part of the diet of some lizards too (Seely & Louw, 1980; Nagy *et al.,* 1991). The dominant invertebrate detritivores vary from desert to desert (Crawford, 1979), and a rationale for the phylogenetic variation is not yet clear. Many taxa of detritivores are inconspicuous because they are confined to the sand. They include species of *Zygentoma* (silverfish), which are ubiquitous in deserts, species of nematode (Treonis *et al.,* 2022), species of mites, the larvae of *Diptera* (flies) and, particularly in Australian deserts, species of *Collembola* (springtails; Crawford, 1979). Termites are extraordinarily abundant detritivores in the Chihuahuan (Whitford *et al.,* 1982), Sonoran (Crawford, 1979) and Australian deserts (Smith & Morton, 1990). Unlike most inhabitants of desert burrows, termites forage far from their nests and so can feed on detritus on nearby loose sands.

The Namib Desert is special, and possibly unique (Seely, 1979*b*), for the abundance of detritivores that are resident on sand dunes that are devoid of vegetation (Lawrence, 1959). The wind regime of the Namib dunes is particularly favourable for the delivery of detritus from the east (Seely, 1983). Furthermore, the detritivores of the Namib have had longer to evolve than have those of other deserts (Ward *et al.,* 1983). The most conspicuous of the detritivores are the adult tenebrionid beetles. That the dominant detritivores are beetles, rather than termites, isopods or millipedes, is unusual for a desert, though isopods and millipedes are excluded from deserts of loose sand (Seely & Louw, 1980). The larval beetles, resident below the sand surface, also are detritivores.

Detritus may be the only source of cellulose for those invertebrates that are confined to the sand, like beetle larvae, but other invertebrates that are active on the surface will use other food sources when they are available. When plants do persist in hot shifting-sand deserts, most of the food that is available to herbivores is above the surface (Hadley & Szarek, 1981). The consumer is required to cope with low digestibility and sometimes chemical defence mechanisms (Smith & Morton, 1990) or to select the more-palatable products of those plants (Curtis, 1985). There is one herbivorous lizard of the Namib Desert, *G. skoogi*, for which the palatable part is a spine. Of the organic matter in its faecal pellets, 55% consisted of remnants of the impenetrable nara plant (Pietruszka *et al.,* 1986; see Fig. 2). Analysis of the stomach contents of adult *G. skoogi* showed that 70% of dietary dry mass consisted of spines of the plant (Nagy *et al.,* 1991). Though those spines are rigid and dangerously sharp, their organic matter is mostly soluble, rather than insoluble like cellulose. Growing spines contain 80% water and the lizards excrete copious urine, implying that they are not hypohydrated (Pietruszka *et al.,* 1986). Nor are the herbivorous chuckwallas *Sauromalus ater* of rocky arid areas in North America (Nagy, 1988).

While *G. skoogi* is endemic to the northern Namib dune sea, the nara plant occurs throughout the Namib Desert. The lizard hatchlings are detritivores (Mitchell *et al.,* 1987). Important questions to be answered, if the species is to be conserved, is how it came to be so isolated, why they do not occur in the Central Namib dune sea that is only a few hundred kilometres away and replete with nara, and why no other Namib Desert lizards eat nara spines.

Other plants also persist on some Namib dunes, especially after rain, and the adult tenebrionid beetles will eat material from those plants, including their seeds (Seely & Louw, 1980). Indeed, virtually all of the herbivores that graze on the plants of shifting-sand deserts are invertebrates (Hadley & Szarek, 1981). Mammals make hardly any impact on the plant biomass in any desert (Hadley & Szarek, 1981; Morton, 1985), lizards only a minor effect (Hadley & Szarek, 1981). While the impact of birds is little known, in the Monte Desert of Argentina (a shrubby rather than sand-dune desert), heavy predation of grass seeds by birds was insufficient to reduce seedling recruitment (Marone *et al.,* 2008). In the Chihuahuan and Sonoran deserts, termites account for at least half of the disappearance of plant litter, including leaves of the bitter creosote bush *Larrea tridentata* (Crawford, 1979; Whitford *et al.,* 1982, see Fig. 2). Apparently more effectively than do birds, they also remove the seeds that would have been responsible for the future propagation of the plants (Hadley & Szarek, 1981; Morton, 1985). Ants also consume plant material, and colony densities of up to 4000 colonies per hectare have been counted in the Chihuahuan Desert (Hadley & Szarek, 1981).

Termites are surprisingly vulnerable to hot dry weather, and their numbers decreased massively in recent hot droughts in the Kalahari Desert (Weyer *et al.,* 2020; Panaino *et al.,* 2023). What would be the consequence for the other residents of shifting-sand deserts if termites failed to clear the plant litter? Also, some desert plants, notably desert fluff-grass *Dasyochloa pulchella* (formerly *Erioneuron pulchellum*, see Fig. 2) in the Chihuahuan Desert (Whitford *et al.,* 1982), do not thrive when termites are absent, presumably because the termites generate or distribute nutrients or the seeds. It may not be immediately apparent to conservationists that the conservation of a desert ecosystem may rely on the conservation of its termites.

The larger animals that are resident in shifting-sand deserts tend to be carnivorous, not herbivorous, but not all carnivores are large. Arachnids, for example, are strict carnivores (Hadley & Szarek, 1981). Few, if any, other taxa are pure carnivores; most species that consume other animals are omnivores that also consume some plant matter (Stebbins, 1944; Hadley & Szarek, 1981), including detritus. Because food is so scarce in shifting-sand deserts, it could be expected that its resident omnivores would be generalist feeders that can make use of all of the nutritional resources that are available (Nevo, 1979), and most are. The lizard *Meroles* (formerly *Aporosaura*) *anchietae*, which is the dominant vertebrate of the dunes of the central Namib Desert (Robinson, 1990), is an exemplary generalist. It can live completely independently of plants, or rely heavily on plants, depending on the local habitat. In the central Namib dune fields, its stomach contents consisted almost entirely of seeds, but at other sites in the Namib the populations eat primarily kelp flies or weevils (Louw & Holm, 1972).

It is difficult even for ectotherms to obtain sufficient metabolic energy in resource-poor shifting-sand deserts. For a lizard that needs to catch invertebrate prey, like those kelp flies, is it better to be an active forager or a sit-and-wait forager? For active foraging to be profitable, the forager needs to acquire prey that contains sufficient energy and water to compensate, and indeed to overcompensate, for the extra energy demand and water loss that is associated with the foraging activity. The profitability, or not, of active foraging depends on more than just the habitat because active foragers and sit-and-wait foragers often are sympatric. In the Colorado Desert of western North America, the zebra-tailed lizard *Callisaurus draconoides* sits and waits; it moves for less than 2% of its 10-h daily activity period. The sympatric western whiptail *Aspidoscelis* (formerly *Cnemidophorus*) *tigris* is an active forager; it moves for 91% of its 5-h activity period. The daily metabolic rate, measured with the doubly labelled water technique, was 54% higher in the western whiptail than it was in the zebra-tail (Anderson & Karasov, 1981). Both ate insects and other invertebrates, but the foraging efficiency (metabolizable energy intake/energy expended) of the western whiptail was 2.0, but only 1.5 for the zebra tail (Anderson & Karasov, 1981)*.* For the western whiptail, active foraging was profitable. The Husab sand lizard *Pedioplanis husabensis* of the Namib Desert, an active forager that eats mainly termites and moves for 50% of its activity period, has a different strategy to attain a foraging efficiency that is favourable. It forages at low energy cost; its daily field metabolic rate was typical for that of sit-and-wait foragers of the same size based on regression analysis (Murray *et al.,* 2014). Active foragers in deserts could reduce energy costs by feeding on congregated prey, such as termites and ants, so one would expect the proportion of social prey in the diet to increase with aridity, and it does so in lizards (Pianka, 1986).

If obtaining sufficient metabolic energy in a shifting-sand desert is difficult for ectotherms, it is even tougher for an endotherm that has a mass-specific metabolic rate an order of magnitude higher. It is thus no surprise that shifting-sand deserts host few resident endotherms, though they can have many mammalian and avian temporary visitors. Amongst the few endothermic residents are the sand-swimming moles on two continents (Figure 2 of Mitchell *et al.,* 2026), the presence of which implies that they can obtain enough energy from the shifting-sand deserts. Where do they obtain that energy, and what are the consequences for their conservation? Excavation sampling, to a depth of about 150 mm, of the shifting-sand habitats of the Namib golden moles showed that 50% of potential invertebrate prey items were *Zygentoma* and 28% were *Coleoptera* (beetles) (Fielden *et al.,* 1990*a*). Analysis of the stomach contents of the golden moles revealed that they ate many different invertebrate species, but 98% of the items in their stomachs were termites. Therefore, the golden moles ignored much of the prey that was available and were highly selective feeders for the termites. The termites occurred in high concentrations around the isolated clumps of dune vegetation, notably the grass *Stipagrostis* (formerly *Aristida*) *sabulicola* (Holm, 1971, see Mitchell *et al.,* 2026).

In contrast to the Namib golden mole, the southern marsupial mole *N. typhlops*, of the shifting-sand deserts of central Australia, does not seem to like termites, but will eat them. The moles are extremely rare, following devastating hunting for their pelts in the 19th Century, and the feeding of free-living moles has not been studied. The intestinal contents from 16 museum specimens that were collected between 1890 and 1910 were examined (Pavey *et al.,* 2012). Ants (mostly adult workers) accounted for 37% of the contents by volume, and beetles (mostly larvae) for 31%, amongst many other invertebrate species. Termites accounted for just 10%, but the habitat abundance of termites was about 35%, implying that the moles should encounter many more termites than they eat. That they do not rely heavily on termites may protect marsupial moles from the consequences of global warming to which termites are very vulnerable (Weyer *et al.,* 2020; Panaino *et al.,* 2023). Given that those marsupial moles forage underground, it was surprising that there were no invertebrate eggs in their intestines. It may be that they are unable to locate underground food items that are inert. Ant eggs may occur in the diet of *N. typhlops*, perhaps when the moles have been able to locate ant nests (Winkel & Humphrey-Smith, 1988).

The avoidance of termites by marsupial moles must be for reasons other than their nutritional value. Table 1 shows the energy and water content of some insects that were collected in the Kalahari and Namib Desert, southern Africa, and in the Australian deserts where the marsupial moles live. The energy content varied little between the insect species, but there were large differences in energy per individual insect because of differences in mass. Eating a single large termite would provide substantial energy (depending on digestibility, Cooper & Withers, 2004) and as we shall see (Section ‘The acquisition and management of water’), sometimes enough water, so that a desert vertebrate that eats termites would not need ever to drink.

**Table 1:** Energy and water content of some potential insect prey of vertebrates of shifting-sand deserts (constructed from data in Robinson (1990), Cooper and Withers (2004) and Weyer *et al.* (2020))

**Species**	**Wet mass (mg)**	**Energy per individual (J)**	**Energy content (kJ**·**g** ^**−1**^ **dry mass)**	**Water mass per individual (mg)**	**Water mass (% wet mass)**
Africa					
*Hodotermes mossambicus* (termite)	17	81	18.0 ± 0.6	13	76
*Trinervitermes trinervoides* (termite)	1.9	14	21.1 ± 0.2	1.2	63
*Anoplolepis custodiens* (ant)	3.2	39	23.9 ± 0.4	1.5	47
*Onymacris sp.* (beetle larvae)			26.9		73
*Zygentoma* (silverfish)			23.4		79
*Comicus sp.* (dune cricket)			26.7		76
Australia					
*Coptotermes sp.* (termite)	1.2	6	23.1 ± 0.2	1.0	78.0 ± 0.4
*Nasutitermes sp.* (termite)	1.2	6	22.7 ± 0.4	0.9	77 ± 3

### The cost of living in a shifting-sand desert

The cost of living in a desert, for all animals in which it has been measured, is lower than the cost of living for similar animals that are resident in less-arid environments (Norris & Kavanau, 1966; Bartholomew *et al.,* 1985; Schwimmer & Haim, 2009). The lower costs can be more dramatic in some shifting-sand deserts than in others; Namib Desert spiders have a metabolic rate that is one quarter of that of similar spiders in other deserts (Henschel, 1997). Sometimes, those who have reported that lower cost of living have expressed the cost in terms of basal metabolic rate (Contreras & McNab, 1990; Akin *et al.,* 2024). The basal metabolic rate is a laboratory phenomenon with biochemical implications, but with little relevance to conservation physiology (Mitchell *et al.,* 2018), and so is an inappropriate measure to compare the costs of living in natural habitats. The measure that is pertinent is the field metabolic rate, the energy cost that is incurred by animals going about their elective activities in their natural habitats (Nagy, 1987, 1994). The field metabolic rate is associated positively with basal metabolic rate, but the relationship is too variable to allow field metabolic rate to be predicted from basal metabolic rate (Contreras & McNab, 1990). The field metabolic rate includes the cost of tissue maintenance, growth, reproduction and obligatory activities like foraging.

**Figure 3:**
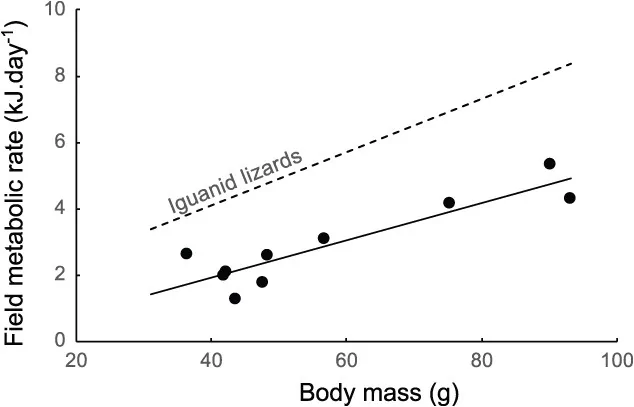
Field metabolic rate, measured with doubly labelled water, of sand-swimming lizards *G. skoogi*, living free in Namib Desert sand dunes (black circles and solid line), compared with that of iguanid lizards over the same mass range (broken line). Redrawn from Nagy *et al.* (1991), by permission of John Wiley and Sons.

The field metabolic rate is measured best with the doubly labelled water technique. Nagy *et al.* (1991) used that technique to measure the field metabolic rate of sand-swimming lizards *G. skoogi* (see Fig. 2) that were living free in their natural habitat of the northern dune sea of the Namib Desert. The mean field metabolic rate of 3.0 kJ·day^−1^ was about half of that of iguanid lizards of similar mass that were measured in other habitats (Fig. 3). The field metabolic rate of Namib golden moles in their natural habitat, measured by Seymour *et al.* (1998) with doubly labelled water, was 12.5 kJ·day^−1^, also about half of that of insectivorous mammals of similar mass that are resident in less-arid habitats.

The low cost of living of the animals of shifting-sand deserts usually is considered to be an adaptation that matches the low energy availability in those deserts (Nagy *et al.,* 1991). Desert animals may have a ‘metabolic switch’ that curtails metabolism when food is scarce and increases it when food is plentiful, without a concomitant change in body temperature or activity level (Merkt & Taylor, 1994). It seems to us, though, that a hypothesis that metabolic rate is matched to food availability is an incomplete explanation of the low cost of living. Either the animals that live in less-arid environments are profligate in their use of metabolic energy or the animals of shifting-sand deserts must sacrifice some physiological function or functions to accommodate their lower energy budget. Desert animals that aestivate or hibernate may sacrifice body mass (Dawson *et al.,* 1989; Cloudsley-Thompson, 1995). What is sacrificed by active animals is not obvious, but we suspect that it may be related to reproduction, with consequences for their conservation.

### The cost of moving in and on sand

To translocate, terrestrial animals that are resident in the deserts with substrates of loose sand have the option to travel on the sand surface or to swim through the sand. Even though it seems counterintuitive, sand-swimming requires less metabolic energy that does burrowing. Sand-swimming is much more costly, though, than is surface locomotion, so when an animal chooses to swim through sand rather than to use the surface, as do foraging marsupial moles, the incentive must be strong.

Whether travelling on the sand or swimming through it, none of the vertebrates of shifting-sand deserts engages in endurance exercise. Rather, they move in short intermittent bouts, each of which can be intense, with extended rest periods between the bouts (Gleeson & Hancock, 2002). Such activity patterns are characteristic of animals with low aerobic capacity (Beck *et al.,* 1995). Oxygen consumption during those short exercise bouts is not an accurate measure of the cost of the activity because there is a substantial excess of oxygen consumption after exercise that restores equilibrium after anaerobic activity (Gleeson & Hancock, 2002). Also, while some birds and mammals that visit the loose sand may use saltatory bipedal translocation (‘hopping’, Eisenberg, 1975), none of the legged residents of shifting-sand deserts appears to do so. Although there are many rodents of firmer deserts that use bipedal hopping, there is no evidence that hopping evolved in deserts (McGowan & Collins, 2018). Hopping is particularly advantageous at higher speeds of locomotion (Irschick & Higham, 2015).

Again counterintuitively, loose sand turns out to be a beneficial substrate for the locomotion of small light animals that engage in short bouts of surface activity (Tulli *et al.,* 2012). The mean speed of species of South American lizards when they ran on sand was about 2.4 m·s^−1^ (nearly 9 km·h^−1^) and it did not matter whether the natural habitat of the species was sand, rock or another terrestrial substrate (Tulli *et al.,* 2012). Although they do not run on sand as fast as do similar-sized Australian tiger beetles *Rivacindela hudsoni*, which run at the same speed as do those lizards (Kamoun & Hogenhout, 1996), the tenebrionid beetles *Onymacris plana*, of the Namib Desert dunes, run at 0.9–1.2 m·s^−1^, extraordinarily fast for a terrestrial insect of 1 g mass (Nicolson *et al.,* 1984). Its cost of running, measured on a treadmill, increased linearly with running speed at low speeds but reached a plateau of about 5.3 ml·O_2_·g^−1^·h^−1^ (about 30 mW·g^−1^) at about 0.13 m·s^−1^; its efficiency at higher speeds may depend on its flattened body that can act as an aerofoil (Bartholomew *et al.,* 1985).

The cost of sand-swimming does not appear to have been measured in beetles or in lizards, but it has been measured in Namib golden moles and in marsupial moles, as well as in the spadefoot toad *S. hammondii* (Seymour, 1973*b*)*.* The measurements made in the toad are credited with being the first measurements of the energy cost of digging in a substrate for an animal (e.g. Wu *et al.,* 2015). The resting metabolic rate of wild-caught toads (mass about 12 g) varied hardly at all with temperature between 7.5 and 32.5°C, but the cost of submerging in sand increased 3-fold between 10 and 30°C, reaching 0.5 ml·O_2_·g^−1^·h^−1^ (about 3 mW·g^−1^) at 30°C (Seymour, 1973*b*). Submergence was undertaken in very short bouts of activity. At all temperatures, the depth achieved at each bout was a tiny 1 mm, approximately, but the toads could retreat to a depth of 160 mm in 30 min.

Twenty-five years after he made those measurements in toads, Seymour and new colleagues measured the cost of sand-swimming, and of surface running, in Namib golden moles (Seymour *et al.,* 1998). The maximum running speed that was reached on blotting paper in a rotating drum was about 0.3 m·s^−1^ (about 1 km·h^−1^), at which speed the rate of oxygen consumption was about 4 ml·O_2_·g^−1^·h^−1^ (about 20 mW·g^−1^). The cost of transport (J·m^−1^) was similar to that of other running mammals of the same mass, despite the morphology of the golden mole apparently not being ideal for running, with short limbs and large claws. The cost of sand-swimming was measured by partially filling the drum with Namib Desert sand. The drum was rotated to keep the mole stationary in space while it swam, at the pace chosen by the mole. Even though the sand in the rotating drum was loosened artificially, the gross energy cost of sand-swimming (80 J·m^−1^) was 26 times higher than the gross energy cost of running. That cost is the equivalent of the metabolic energy contained in one *Hodotermes* termite or two *Anoplolepis* ants for each meter traversed. The moles could maintain sand-swimming at a speed of up to 0.01 m·s^−1^ (0.04 km·h^−1^), but no higher. Therefore, a golden mole that travelled 4 km in a night (Holm, 1971) could not have spent much time swimming below the sand surface. Indeed, Seymour *et al.* (1998) estimated that a foraging golden mole would spend only 1% of its time below the sand.

Seymour and colleagues had access to 14 Namib golden moles that were captured, studied and then released at the capture site on the same day. While the golden moles are rare and difficult to catch when they are buried in the desert sand, ‘marsupial moles are extremely rare and it is almost impossible to capture specimens alive’ (Withers *et al.,* 2000, p. 242). When Withers and his colleagues made measurements on marsupial moles, they had access to just a single 34 g specimen of *N. caurinus,* which survived in captivity for 5 weeks (Withers *et al.,* 2000). The energy cost of running and sand-swimming was measured in the same way that they had been for the Namib golden mole. On the surface, the mole ran for more than 40 min ‘with a slow and seemingly laborious “shuffling” action’ (Withers *et al.,* 2000, p. 245) at a speed of 0.13 m·s^−1^ (range 0.09–0.19 m·s^−1^), so more slowly than did the golden mole at maximum speed. The speed that was maintained during forced sand-swimming was much more variable, with a maximum of 0.005 m·s^−1^, half that of the Namib golden mole. The gross energy cost of transport during forced sand-swimming was 550 J·m^−1^, much higher than the cost for the Namib golden mole and 60 times higher than its own gross cost of transport by running. However, marsupial moles do not forage on the surface and so apparently do not take advantage of that lower cost of running.

**Figure 4:**
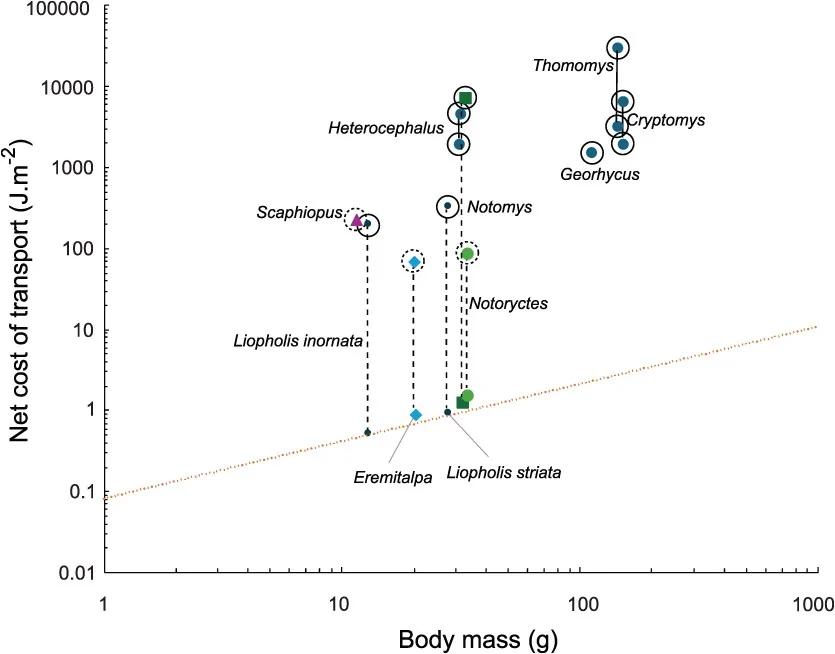
Net cost of transport of some vertebrates while they were sand-swimming (symbol surrounded by dashed circle), burrowing (symbol surrounded by solid circle) and pedestrian (naked symbol) as a function of body mass. The sand-swimmers are the Namib golden mole *E. granti namibensis,* the northwestern marsupial mole *N. caurinus* and the spadefoot toad *S. hammondii*. The burrowing mammals are species of mole rat (*Heterocephalus, Georychus, Cryptomys*), the pocket gopher *Thomomys bottae*, and the spinifex hopping mouse *Notomys alexis*. Data also are shown for two species of burrowing skink from Australian deserts, *Liopholis striata* and *Liopholis inornata.* The net cost of transport also is shown for the golden mole, marsupial mole, hopping mouse and two skink species in pedestrian mode, and a regression line for running animals over the mass range. Solid lines join measurements on the same species in the same locomotor mode, dashed lines join the same species in different locomotor mode. There is nothing unusual about the net cost of transport in the moles and skinks when they were running, or the mice when they were hopping, compared to the cost of walking and running in the other pedestrian animals. The cost of sand-swimming (voluntary for the toad, but forced for the moles) was similar for the three diverse species and much higher than the cost of transport when running in those species. The cost of sand-swimming was at least 10-fold lower than the net cost of transport when burrowing. The cost of burrowing in the skinks was less than that of the mammals but more than 300 times their cost of running. Calculation of the net cost of transport requires deduction of the resting metabolic rate from the gross cost of transport. Reconstructed from Figure 5 of White *et al.* (2006), with the value for the spadefoot toad recalculated from Table 5 of Seymour (1973*b*). Data for the marsupial mole from Withers *et al.* (2000) and for the skinks from Wu *et al.* (2015).

Figure 4 shows the net cost of transport for animals of varying size. Despite their anatomy being quite different to that of an athletic animal (see Fig. 2), the net cost of transport of the golden mole and marsupial mole while running was close to the regression line for the other animals, as was the cost for the hopping mice when they were hopping. The cost of sand-swimming in the mammals was much higher than the cost of pedestrian movement, but not nearly as high as the net cost of transport for burrowing. In Appendix 3, we explore why the cost of burrowing is higher than is the cost of sand-swimming.

What lessons relevant to conservation physiology can be learned from the turnover of metabolic energy by the animals of shifting-sand deserts? Amongst those lessons is that evolution has helped those animals to contribute to their own conservation through selection for a field metabolic rate that is much lower than the field metabolic rate of animals that have access to more nutrition. It is unlikely that there are no functional penalties to pay for the frugal energy intake and expenditure, but what those penalties are remains unknown. For their energy, the animals of shifting-sand deserts are heavily reliant on cellulose as a source, and their conservation will require that there are no barriers to the supply of cellulose, particularly in the form of detritus. Burrowing is the most expensive mode of terrestrial translocation, so the fact that it has evolved numerous times in animals that live in resource-poor habitats (Marchetti *et al.,* 2024) implies that the benefits, for example, escape from predation or from adverse weather, must be substantial. Living fully or partially below the ground surface does seem to benefit the mammals that live in the harsh habitat of a shifting-sand desert and appears to have done so over evolutionary time (Pinkert *et al.,* 2025).

## The acquisition and management of water

One would expect that the animals that live in shifting-sand deserts have adapted to cope with the dryness that defines a desert because they have survived there. As Vernon Bailey, then Chief Field Naturalist of the US Department of Agriculture, said more than 100 years ago: ‘Even in the midst of long dry periods these desert animals are found in perfect health and good bodily condition, with abundance of internal fluids and secretions’ (Bailey, 1923, p. 66). Some species living in less-arid habitats have adapted to seasonal dryness via migration. The ungulates of the Serengeti and Maasai Mara in Africa, for example, migrate away from relative dryness to where the annual rains will fall. In their habitat, the seasonal rainfall is reliable and predictable. The adaptations of animals in shifting-sand deserts cannot depend on any seasonal clock because not only is precipitation rare in shifting-sand deserts, but it is unpredictable and tends to be localized (Crawford & Gosz, 1982; Smith & Morton, 1990). What the animals in shifting-sand deserts have adapted to do is to persist through periods when they have no access to free water, with no information about when that period will end.

When that period does end, the free water that becomes available can be in the form of river flow, rain, dew or fog, and when droplets fall or form, the water can be in abundance. Animals may choose to drink that water only when hypohydrated, a situation where body water content already is below the ideal. That strategy is dangerous if access to water is unpredictable. An attractive alternative strategy for desert animals would be to acquire as much water as is possible when it is available and to store water in their bodies, physically or biochemically. Some desert animals indeed do just that (see Section ‘Osmoregulation’ below).

When animals do not have access to adequate water and so dehydrate, they do so slowly. The physiological processes that have evolved to deal with the hypovolaemia and hyperosmolality that will result when hypohydration develops have to counteract a gradually increasing threat. If the animals consume a substantial quantity of fresh water when it becomes available, they are at risk of an osmotic shock on themselves, in the form of rapid dilution of body fluids. Unchecked, water will flow from the diluted body fluids into cells, causing those cells to swell and even rupture. Successful desert animals have evolved physiological mechanisms that prevent or alleviate that shock of rehydration (see Section ‘Osmoregulation’).

### The acquisition of body water in shifting-sand deserts

Perhaps counterintuitively for those who are unfamiliar with sandy deserts, many sandy deserts have large rivers that flow through them or along their edges. Some, like the Nile, are permanent rivers and so provide uninterrupted drinking water to any animal that can reach its banks, but many rivers of sandy deserts are ephemeral. Their headwaters lie outside the desert and the rivers carry water only when they flood, after rainfall in those headwaters. The rivers provide a linear oasis of vegetation through their deserts, but they do not continually supply drinking water to animals. On the contrary, many may not flow at all during the lifetime of a desert animal.

Even when rain does fall in a shifting-sand desert, it does not usually provide standing water. Rain can fill endorheic pools (basins that have no outflow to external bodies of water) on impermeable intrusions in the shifting-sand deserts, like rocky outcrops, but those pools are temporary. Rain that falls on the sand does not pool but does penetrate the sand, increasing the humidity below the surface (Robinson & Seely, 1980), which, as we shall see, is life-saving for some animals that live below the surface. Because rainfall in a shifting-sand desert is scarce, intermittent and unpredictable, some plants have evolved mechanisms that confine their reproduction and growth to periods that follow relatively heavy rainfall. They are attuned to the occurrence of rain in pulses, which it typical in deserts (Ward, 2016). In the extreme, in the Namib Desert, some grasses survive with reproduction and growth only every 40 years (see Mitchell *et al.,* 2020). No animal can alter its metabolism and activity in that way. In deserts where some rain falls annually, desert reptiles that are frugal with water can survive on it; the Gila monster *H. suspectum*, of the deserts of North America, is an example (Gienger *et al.,* 2014). However, when the animals of shifting-sand deserts do acquire water from open sources, that source is usually not rain but non-rainfall precipitation, in the form of fog and dew.

However, not all the animals of shifting-sand deserts require free water. In arid Australia, none of thousands of invertebrate species, nor hundreds of reptile species, need to drink, and only 5% of mammal species need to do so (Smith & Morton, 1990). The animals that do not need to drink can survive on metabolic water and the preformed water in food. The provision of access to open water to such animals in the interests of their conservation would be a misdirected activity. Commendably, the Saudi authorities provide no artificial source of water in the 220 000 ha Mahazat as-Sayd Protected Area, an area that has no natural source of permanent standing water but supports large mammals (Hetem *et al.,* 2010).

Every animal makes water during the oxidative metabolism of carbohydrate, fat and protein. The metabolism of cellulose is described by the equation (C_6_H_10_O_5_)_n_ + nO_2_ = 6nCO_2_ + 5nH_2_O. Five molecules of water are synthesized for each saccharide fragment oxidized, with five oxygen molecules consumed, implying metabolic cost. The mass of water that is produced depends on the substrate that is oxidized, at 0.67 g·L^−1^ O_2_ for the metabolism of cellulose and 0.53 g·L^−1^ O_2_ for the metabolism of fat (Morrison, 1953). As well as the synthesis of water, the metabolism of stored glycogen releases bound water into the body fluid space (Sinclair *et al.,* 2024). While the substrate is relevant, a more important factor in the production of metabolic water is the metabolic rate. Any increase in the general level of activity, and particularly flying in insects, increases the metabolic rate massively and therefore also the rate of production of metabolic water. Nevertheless, the mating flights of the males of the digger bee *Centris caesalpiniae*, found in the Sonoran and Chihuahuan Deserts, seem to be limited by dehydration (Johnson *et al.,* 2023). In the hyperarid Atacama desert, water availability interfered with the diurnal activity pattern that tended to be bimodal, and affected the body temperature, of adult tenebrionid beetles (Tirado *et al.,* 2018).

Small animals, including arthropods, rely more on metabolic water than do larger animals to balance their water budget (Wigglesworth, 1946). The question has arisen of whether, in times of relative plenty, desert arthropods lay down fat not just as a reserve of energy, but as a source of metabolic water when water in the environment is in particularly short supply. It is an attractive idea, and the profit/loss calculations are not straightforward because the metabolism of fat requires oxygen and therefore involves an increase in respiratory ventilation and its associated water loss (see Section ‘The retention of body water in shifting-sand deserts’). In only one of five species of tenebrionid beetles from the Namib Desert did fat metabolism compensate for dehydration (Mitchell *et al.,* 2020).

For most of the animals in shifting-sand deserts, preformed water contributes much more to water balance than does metabolic water. Preformed water is liquid water that is contained within, or attached to, the food that an animal eats. Food deficiency in animals that depend on preformed water will lead not only to starvation but also to hypohydration. When the food is animal prey, the bulk of the water will be in the tissues of the prey; more than 75% of the mass of a termite is water (see Table 1). When the food is dry plant material, as is detritus, water is attached and potentially absorbed if the material is hygroscopic (see next paragraph). The attached water may be almost pure, whereas the water in tissues contains electrolytes and other solutes (Clarke & Nicolson, 1994). As pioneering desert biologist Buxton (1923) discovered, the quantity of preformed water in and on otherwise dry plant material can be considerable if the material has been exposed to high concentrations of water vapour, and especially to fog or dew. How plant material accumulates water from the atmosphere is described in Appendix 4.

When preformed water in the diet is in animal prey rather than in plant material, the consumption of enough prey to fulfil energy needs usually also fulfils water needs. The most prevalent of those animals that fulfil their water needs in that way in shifting-sand deserts are carnivorous and omnivorous lizards. Unlike desert scorpions, which waste much of the water in their prey and their own water because the prey is dismembered and partially digested by fluids that are ejected by the scorpion before the prey is ingested (Hadley, 1974; Yokota, 1984), lizards consume all of the water in their prey. Whether or not they can maintain water balance on that prey depends on how much irrecoverable water they need to use to digest it (Chabaud *et al.,* 2023). On average, the omnivorous lizard *M. anchietae*, of the Namib Desert sand dunes, needed 89 mg of dry seeds per day to fulfil its energy needs, from which it derived 9 mg of preformed water. If instead it consumed just one larva of a tenebrionid beetle, which would provide 210 mg of preformed water, or 23 times as much water as did the seeds, as well as four times as much energy (Robinson, 1990). When it consumes enough termites to satisfy its energy requirements, the Husab sand lizard *P. husabensis*, also of the Namib Desert, acquires preformed water in excess of its needs (Murray *et al.,* 2014). In captivity, the Mojave fringe-toed lizard *Uma scoparia*, found on sand dunes of the deserts of north-west America, can survive on a diet of tenebrionid larvae without access to drinking water, and access to water did not change the body mass from that observed on food alone (Munsey, 1972), but not all desert lizards can survive on metabolic and preformed water. The diurnal gecko *Rhoptropus bradfieldi*, which is sympatric with *P. husabensis* but with a preference for rocky substrate, cannot maintain water balance on its diet of ants, according to doubly labelled water analysis (Murray *et al.,* 2014). The lizard *U. scoparia* did drink avidly if it was offered water in captivity (Munsey, 1972). In their natural habitats, other desert lizards do drink if free water happens to become available, after rain (Munsey, 1972). Knowledge of which lizard species need to drink clearly is important for their conservation.

It is not just desert ectotherms that can survive on metabolic and preformed water, without free water. In captivity, the Namib golden mole *E. granti namibensis* survived well without access to drinking water on a diet of tenebrionid larvae, though its daily water turnover was very low compared with that of other insectivores in captivity (Fielden *et al.,* 1990*c*). About three-quarters of its water intake was derived from preformed water and one quarter from metabolic water. It ignored drinking water if it was offered.

In shifting-sand deserts that experience fog, for the animals that do need to drink, or which will drink opportunistically if free water is available, the most common source of free water is likely to be that fog. The phenomenon has been studied in detail only for the Namib Desert (Mitchell *et al.,* 2020). Although much more frequent than rain, the arrival of fog on the dunes of the Namib Desert is highly unpredictable, with the maximum recorded interval between fogs, at different distances from the coast, varying from 33 to 772 days (Lancaster *et al.,* 1984). None of the other foggy deserts has the combination of cool coastal climate, large sand dune area and gentle rise from the coast that the Namib has (Mitchell *et al.,* 2020), but fog is likely to play a role, in the provision of drinking water, at least in the shifting-sand deserts of Baja California (Hiatt *et al.,* 2012), Oman (Ghazanfar, 2004), Yemen (Noman & Al-Jailani, 2007) and western Sahara (Żmudzka *et al.,* 2014). There is very little known about the physiology of the animals in those other fog-dependent shifting-sand deserts, a situation that must hamper conservation decisions. Fog occurs in the Negev desert, but dew is the more-usual source of free water there (Kidron, 1999). The ecology of the Atacama Desert is highly dependent on fog (Cáceres *et al.,* 2007), but it is not a shifting-sand desert.

In the Namib Desert, 48 species of animal are known to, or are highly likely to, use fog water (Mitchell *et al.,* 2020). Those 48 mostly are invertebrates. The vast majority use fog opportunistically; they will drink from fog droplets if they encounter those droplets while they are going about their normal business. However, a few beetle species are active fog harvesters. They will seek out fog water on the desert surface at times of day when they normally would be buried in the sand, and when the ambient temperature is at a level that they would normally avoid (Henschel & Seely, 2008). More details of how they access fog water are given in Appendix 5. None of the vertebrates of any shifting-sand desert uses the intriguing technique of the burrowing thorny devil, *Moloch horridus,* of arid Australia, which captures water in furrows in its skin that is then carried by capillary action to the mouth (Comanns *et al.,* 2016).

Fog droplets form when the temperature of the air falls below the dew-point, that is, the temperature at which the water vapour pressure in the air equals the saturation vapour pressure at that lower temperature. The droplets form in the air, and while water vapour is transparent to visible wavelengths, liquid water is opaque and thus fog is visible. The droplets may or may not deposit on solid surfaces. Dew droplets form when water vapour condenses on a cool surface. That can be on plants, detritus or rocks or on the surface of an animal (Lasiewski & Bartholomew, 1969). Surfaces in sandy deserts usually cool to below the dew-point because those surfaces lose radiant heat to the cold night sky (Beysens, 1995; Zangvil, 1996). Thus, dew does not form under fog because the fog is at the temperature of the air, not at the temperature of the unobstructed night sky. Although dew can form on the sand itself, it rarely persists there (Agam & Berliner, 2006). Dew is measured at weather stations via the measurement of the rate of water condensation on an impermeable surface, so the dew that is reported at a weather station will overestimate the amount of dew that is available to the animals of shifting-sand deserts (Kidron & Starinsky, 2019). After dew condenses on the surfaces of a shifting-sand desert, however, water vapour does increase in the upper layers of the sand (Kaseke *et al.,* 2012).

At Avdat in the Negev Desert (Israel), the amount of dew recorded over a year was about a third of the annual rainfall, but the rainfall was confined to the winter and was highly variable from year to year. The dew was more consistent and occurred throughout the year, but especially in the summer, when it condensed, on average, on two of every three nights and so provided a reliable source of water for resident animals (Broza, 1979). Many arthropod species access that dew (Broza, 1979) as does the desert snail species *Xerocrassa* (formerly *Trochoidea*) *seetzenii* (Degen *et al.,* 1992).

**Figure 5:**
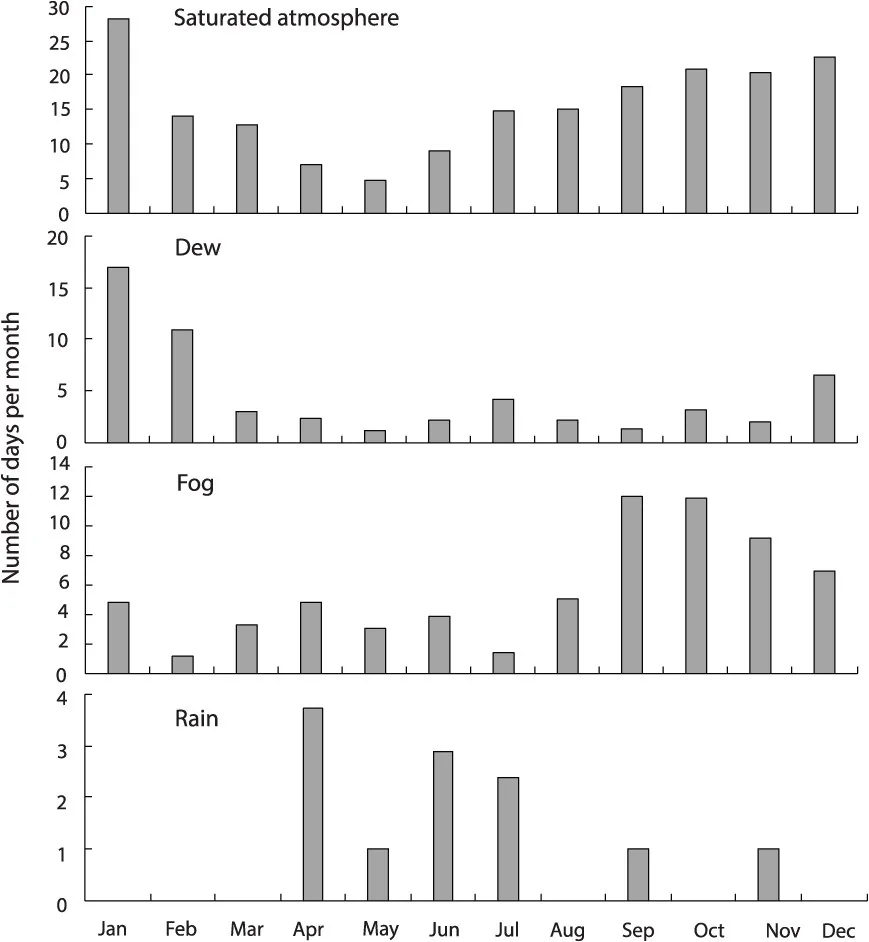
The number of days in each month of the year 2001 on which rain, fog or dew occurred, or on which the relative humidity of the air exceeded 97% (defined as a ‘saturated atmosphere’), at Gobabeb in the Namib Desert. Note the different scale on the *y*-axes for the different forms of water. Reconstructed from Mitchell *et al.* (2020).

As well as rain, dew and depositing fog, there is another source of water that can be available to some of the animals of shifting-sand deserts. It is the water vapour in the air. Though it has been known for a century that insects can extract liquid water directly from the water vapour in the air (Bodine & Orr, 1925), and for almost as long that the air does not need to be saturated (Ludwig, 1937; Wigglesworth, 1946), it remains counterintuitive that an animal can obtain liquid water from the water vapour in unsaturated air. The survival of some animals may depend on them being able to do so, and especially those that are too small (typically those with a body mass <100 mg) to generate enough force to break the surface tension on water droplets (Mitchell *et al.,* 2020). In shifting-sand deserts, and especially coastal shifting-sand deserts, the number of days in a year when the relative humidity is high exceeds by far the number of days on which fog or dew occur (Fig. 5). Even in fog, there is much more water in the vapour phase than there is in the liquid phase (Eugster, 2008). How desert animals obtain liquid water from the water vapour in unsaturated air is described in Appendix 6, and a general overview of how desert animals access body water appeared in Gurera and Bhushan (2020).

### The retention of body water in shifting-sand deserts

Most of the animals of shifting-sand deserts need to retain as much as possible of any water that they acquire, whether it be until the next feed or until the next opportunity to obtain free water. Smaller animals are disadvantaged because they have a large interface with the environment relative to their mass. Especially disadvantaged are small dolichomorphic (slender shape with long limbs) animals like ants; the price that ants pay for protection against solar radiation (see Mitchell *et al.,* 2026) is a massive interface with the environment via which water can be lost. The animals of shifting-sand deserts can use behavioural means to reduce the risk of hypohydration, but those animals also have access to other means to prevent or reduce water loss. The means include an integument that is particularly impermeable to water, mechanisms that recapture as much water as possible from excreta, and mechanisms that limit the rate of evaporation of water into expired respiratory gases. Whatever means that they use, the residents of shifting-sand deserts are remarkably successful in the retention of body water. They have much lower rates of water turnover than do residents of other habitats, and they seldom succumb to hypohydration except in extreme conditions.

**Figure 6:**
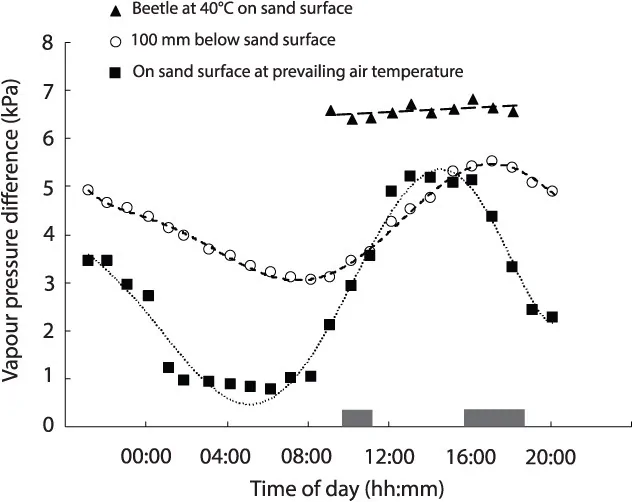
Vapour pressure difference between a hypothetical beetle and its surroundings on the surface and 100 mm below the surface of a sand dune near Gobabeb in the Namib Desert. The temperature and relative humidity of the air were measured at beetle height over the sand surface and 100 mm below the surface. The beetle temperature was assumed to be equal to the temperature of the surroundings. The vapour pressure difference between the beetle and its surroundings was calculated assuming that the beetle had 99% interior relative humidity. The 40°C line designates the vapour pressure difference for a black beetle on the surface and exposed to the sun (Hamilton 1975). The grey bars on the *x*-axis indicate times when the beetles were active on the surface of that dune. Reconstructed from Seely and Mitchell (1987). Smoothed curves were fitted by eye.

We alluded (Mitchell *et al.,* 2026) to the postulate that the main purpose of sand-swimming often is taken to be a means to escape from hostile surface weather (e.g. Dawson *et al.,* 1989), but warned that the assumption that the sand is less hostile than the surface has rarely been explored. It often is taken as a given that the substrate below the ground surface in deserts must be moister than the air above the surface and therefore that the subsurface substrate provides a hygric haven (Eckardt *et al.,* 2013; Pirtle *et al.,* 2019). Walsberg (2000) contested the proposition that burrows necessarily provide a subsurface hygric haven in arid environments, but what about the sand, for animals that swim rather than burrow? To compare the potential for water loss on the surface and below, we measured temperature and relative humidity and derived the water vapour pressure above and below the sand surface across a sand dune transect on the Namib Desert coast (Seely & Mitchell, 1987). Figure 6 shows the vapour pressure difference over 24 h at one of the sites, at which only 5.3 mm of rain had fallen in the preceding year. The plot shows the difference in the water vapour pressure between a beetle, with 99% relative humidity in its tissues (O’Donnell & Machin, 1988), and its surrounds. It is the vapour pressure difference that is the driving force for dehydration (Anderson, 1936; Mitchell *et al.,* 2024; Sinclair *et al.,* 2024), not the difference in relative humidity as is often promulgated incorrectly. Water vapour can move against a gradient in relative humidity (Mitchell *et al.,* 2024).

For most of the 24 h that is illustrated in Fig. 6, a beetle buried under 100 mm of sand, a burying depth that is typical for Namib tenebrionid beetles, would have been under more dehydration stress than would a shaded beetle on the surface. For only about 3 h in the early afternoon was dehydration stress higher on the surface than at 100 mm below surface. Around that time, a beetle in the sun would have been under more dehydration stress than one in the shade or below the surface, but beetles chose to be active on the surface then, despite the dehydration stress. Presumably their intrinsic resistance to water loss (see below) allowed them to be active despite the risk of dehydration. Also, if the avoidance of dehydration stress was the driving force that determined when the beetles were on the surface, they would have been active at night. The results of these calculations do not support the postulate that the beetles sought a hygric haven by burying in the sand. Nor do the results of Holm and Edney (1973), who originally discovered that the sand of the Namib dunes holds very little water vapour. Vernon Bailey was off the mark when, a century ago, he said that desert animals were ‘not exposed to the drying air of the hot days’ (Bailey, 1923, p. 69). In the Namib Desert, at least, they are exposed to drying air and appear unconcerned.

One way that the animals of shifting-sand deserts could avoid exposure to the drying air of hot days indeed is to switch their activity to the night. That would disrupt their social milieu or might expose them to novel predators. If the diet of carnivorous or omnivorous animals consists of diurnally active prey only, they could not make the switch. However, it is a strategy used by desert herbivores that are too large to burrow or to swim in the sand (Hetem *et al.,* 2012; Davimes *et al.,* 2017). Modelling studies have indicated that if the Australian diurnal lizard *Egernia cunninghami* switched its activity to the night, it would reduce water loss by 88% (Pirtle *et al.,* 2019). The fact that so few field studies of the physiology of the animals of shifting-sand deserts have been carried out at night compromises the knowledge base that is required for their conservation.

If they are not to employ such major changes in behaviour to retain water, the animals of shifting-sand deserts require adaptations that will operate both within the sand and on the surface of the sand. One adaptation that has evolved, presumably independently, in many species, is an integument that is highly impermeable to water. For insects, how the impermeable integument is formed and how it functions was described remarkably well by Wigglesworth (1946, p. 210), who observed that the insect ‘cuticle is rigid, flexible or elastic according to the mechanical needs of the system; but it remains extraordinarily impermeable to water’. The impermeability arises from a thin film (<1 μm, O’Donnell, 2022) of wax deposited on the outside surface of the epicuticle. More details about the wax layer are given in Appendix 7.

While an impermeable integument is widespread amongst arthropod species, it is not ubiquitous amongst reptile species, some of which inhabit the same desiccating desert environments as do desert tenebrionid beetles (Munsey, 1972). In other reptiles, cutaneous water loss can be substantial and is thought to have a cooling function (Dantzler & Bradshaw, 2008). The reptiles of shifting-sand deserts have forsaken cutaneous evaporative cooling and have evolved a less permeable integument, implying a degree of flexibility in cutaneous water loss in the class Reptilia. Dmi’el (2001) measured and contrasted the skin resistance to water loss in eight species of snake and five species of agamid lizard that are resident in a mesic to hot desert, ranging from semi-aquatic to fully terrestrial habitat, in Israel. In all of the snakes and the lizards, the skin resistance was higher in desert-dwelling species. Similarly, in North America, the level of cutaneous water loss (mg·cm^−2^·day^−1^) was only one-quarter in the chuckwalla *S. ater* of rocky arid areas of what it was in the green iguana (*Iguana iguana*)*,* an arboreal species of lizard that is often found near water (Dantzler & Bradshaw, 2008). A data base of the rate of evaporative water loss in 301 species of squamate reptile has been compiled more recently, but at this stage a full ecophysiological analysis of the data has not been done (Le Galliard *et al.,* 2021).

It would be counterproductive for an animal of a shifting-sand desert to have an integument that is impermeable to water if it copiously voided water in its excreta. There may be a mechanical reason, but there is no hydroregulatory reason for faeces that are about to be voided to contain water. The cryptonephrideal complex, in insects that have it, can reclaim every trace of water in faeces before those faeces are voided (Wigglesworth, 1946; Beaven & Denholm, 2025). The excretion of urine is complicated by the need to transport excess electrolytes and the soluble products of nitrogen metabolism to the point of excretion, and the animals of shifting-sand deserts require special strategies to retain water when those solutes are excreted. Reptiles do not have the kidney structure that is necessary to concentrate urine; they do not have the loops of Henle that create an osmotic gradient and are responsible for the concentration of urine in mammals (Bradshaw, 1975; Dantzler & Braun, 1980). How the animals of shifting-sand deserts retain water while excreting products of nitrogen metabolism is explained in Appendix 8.

The final route of water loss that is amenable to reduction in animals that need to limit water loss is respiratory water. Water is lost from the respiratory tract whenever the ambient water vapour pressure is lower than the water vapour pressure on the surface of respiratory passages; the inspired air is conditioned by evaporation from the respiratory surfaces into the air (Richards, 1970). In many mammals and birds that physical principle induces evaporative heat loss, which is enhanced if the animal pants. Although lizards are poikilotherms, some do pant under heat load, although the threshold of body temperature at which panting starts is typically higher than 36°C (e.g. Loughran & Wolf, 2023). Also, panting in lizards can cause alkalosis because it is not confined to ventilation of the dead space (Crawford & Gatz, 1974) as it can be in mammals and birds.

**Figure 7:**
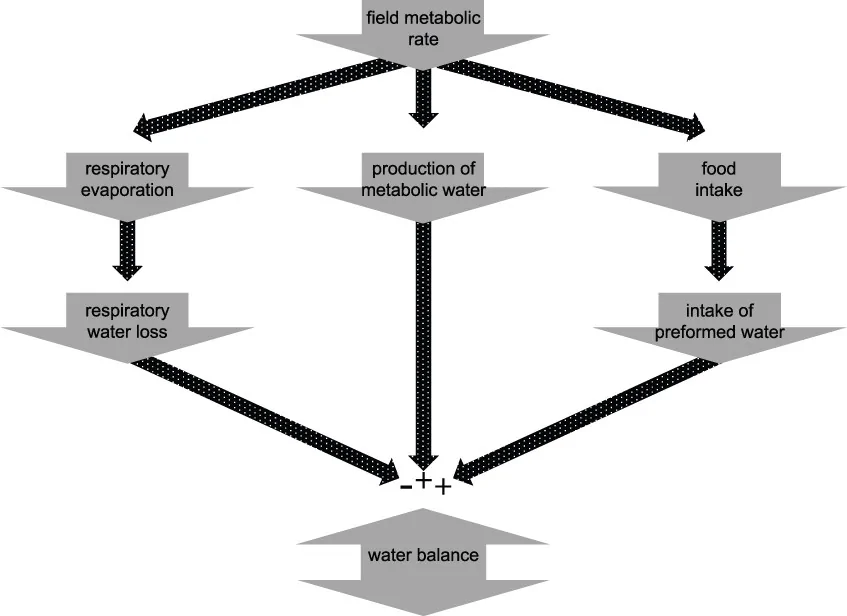
Some consequences of low field metabolic rate for body fluid balance. A lower metabolic rate leads to a lower respiratory water loss but also implies a lower production of metabolic water, and implies that the animal will need less feed and consequently access to preformed water will fall. The outcome for body water balance will depend on the relative size of the three pathways. The diagram does not include the potential effects of a lower field metabolic rate on cutaneous water loss.

The lizards of shifting-sand deserts can substitute conductive cooling to sand for evaporative cooling by panting, but no animal can avoid the addition of body water to gases that are inspired into the respiratory tract for the purpose of gas exchange with the blood or haemolymph. Those tracts can be lungs and airways in lizards, birds and mammals or the spiracles of insects. The relative contribution of respiratory water loss to total body water loss is particularly high in insects from arid environments, probably because selection has reduced the potential for the loss of water via all other routes (Zachariassen, 1996; Addo-Bediako *et al.,* 2001). Respiratory water loss is unavoidable, but a reduction in its rate can be achieved in several ways. One way is simply by a reduction in the flow rate of ventilation, achieved by a lowering of metabolic rate, which is characteristic of many desert species (see Section ‘The cost of living in a shifting-sand desert’). However, a low metabolic rate also reduces the rate of production of metabolic water, so the outcome for body water balance is not straightforward, as the flow diagram of Fig. 7 shows. That the animals of shifting-sand deserts have evolved a low metabolic rate does not necessarily imply that reduced respiratory water loss was the driving force. It was more likely to have been a lower requirement for food, in the resource-poor environments of those deserts, with reduced respiratory water loss as a secondary benefit.

The spiracles of insects, which is their respiratory tract, are not passive openings to the environment but are under active control. Tenebrionid beetles from Arizona, USA, lost water at a much higher rate soon after they were killed than they did when they were alive (Ahearn, 1970). If the spiracles of a beetle are kept open experimentally, the insect desiccates and dies (Wigglesworth, 1946), implying that the spiracles play a role not only in the exchange of respiratory gases, but also in the control of water loss. The air inside spiracles does not exchange gases continuously with the environment, especially in insects like the tenebrionid beetles that have fused elytra. Water loss through spiracles is described in Appendix 9.

We have presented a repertoire of processes by which the animals of shifting-sand deserts could restrict water loss, namely water-saving behaviour, waterproof integument, the elimination of water from faeces and the excretion of the metabolites of nitrogen metabolism in urate and the curtailment of respiratory evaporative water loss. How well can those animals use that repertoire to maintain water balance in their arid habitat? The water economy index ([WEI], ml·kJ^−1^) can be used to compare the use of water by animals with different metabolic rates. The WEI is the ratio of water turned over per day (i.e. water input, including metabolic water, and water lost each day) to the metabolic energy consumed per day (Nagy & Peterson, 1988). It tends to be low in desert animals, conveying an economic use of water relative to the cost of living (Nagy *et al.,* 1991). However, with the caveat that there was no phylogenetic correction in the analysis, the WEI of seven desert reptile species was not different, statistically, to that of six non-desert species, but the desert reptiles had a water turnover rate that was half that of the non-desert reptiles (Nagy, 2004). So those desert reptiles used mechanisms that reduce water turnover that did not rely on any reduction in water turnover relative to energy turnover (see Fig. 7). In captivity with *ad libitum* access to moist vegetable food, the diurnally active Namib lizard *G. skoogi* could balance its water budget at 37°C air temperature. Under those conditions, evaporation accounted for 38% of its water loss and excreta for 62% (Clarke & Nicolson, 1994). Also in captivity, at 30°C air temperature, and fed larvae of tenebrionid beetles, the Namib golden mole could balance its water budget and lost only 25% of its water in excreta but 75% by evaporation (Fielden *et al.,* 1990*c*). Desert invertebrates lose much less water in excreta than do those of less-arid habitats (Ahearn, 1970; Cooper, 1985).

### Osmoregulation

The animals of shifting-sand deserts cannot survive without acquiring and retaining the water and managing their water and solutes balance, and they usually do so successfully. The water content of desert animals is no different to other animals; ‘Desert animals are composed of 65–75% water, just like non-desert animals’ (Nagy, 2004, p. 291), meaning that their adaptation to a lack of water has been in how they manage water gain and loss physiologically.

Desert reptiles can tolerate a water loss of no more than about 25% of body mass (Pirtle *et al.,* 2019). Wigglesworth (1946) thought that an insect could not tolerate the loss of a greater proportion of its body fluids than could other animals, but he was wrong. The tenebrionid beetle *Trachyderma philistina,* one of the species that eats plant material that was wet by dew in the Negev Desert, can lose 35% of its body mass without ill effect (Broza *et al.,* 1976). Though the management of osmolality and electrolyte concentration during the relatively slow process of dehydration is difficult enough physiologically, the management of solutes during rapid rehydration is a much more formidable task. When the Gila monster binge drinks, its body mass can increase by 22% and its plasma osmolality can decrease by 24% (Davis & DeNardo, 2007). The invertebrates of shifting-sand deserts confront even more serious challenges to osmoregulation from the rate of water gain when water becomes available. The mass of the trench-digging beetle *Lepidochora discoidalis* can increase by 41% after a single fog event (Seely & Hamilton III, 1976). The acquisition of such large amounts of pure water has the potential to cause a catastrophic decreases in the concentration of electrolytes, glucose, amino acids and other solutes in body fluids. How insects manage to maintain the membrane potentials of cell membranes during disturbances to electrolyte concentration is a mystery, to us at least.

Some of the animals of foggy deserts have a unique solution to the dilution of solutes during rapid rehydration. They capture the water when they have the opportunity, but do not release it into body fluids immediately. In the laboratory, Namib sand dune lizards *M. anchietae* were first dehydrated and then given access to water. Their plasma osmolality hardly changed after they drank, implying that the water was not absorbed (Louw & Holm, 1972). Those lizards have evolved an unusual gastrointestinal function such that they can store water first in the stomach and then in the caecum, maintaining the osmolality in those compartments lower than that of plasma. How water can traverse the intestine without its osmolality equilibrating with that of body fluids was a mystery to Louw and Holm (1972) and remains a mystery to us. The lizards then release water from the caecum gradually over the next 8 weeks (Louw, 1971; Louw & Holm, 1972; Cooper & Robinson, 1990). The fog-basking Namib tenebrionid *O. unguicularis* (Hamilton III & Seely, 1976; Seely, 1979*a*) was later shown to also have the capacity to store fog water in the gut. The capacity to use the urinary bladder (and not all lizards have a urinary bladder; Beuchat, 1986) as a reservoir for body water was later discovered in the Gila monster; the reabsorption of water from the bladder could buffer plasma osmolality when food and water was in short supply (Davis & DeNardo, 2007). More details of how animals of shifting-sand deserts osmoregulate are given in Appendix 10.

We have related how desert animals acquire water, particularly from their food, from fog and dew, and, surprisingly, from water vapour in unsaturated air. To retain what they require, they need adaptations that operate in sand as well as above it, like integuments that are made impermeable by a 1 μm coating of lipids. They have solved the problems of fluid management via urate pellets and nasal glands that eject more than just sodium chloride (Appendix 10). Unapologetically, we have focussed on the predominant vertebrates of shifting-sand deserts, namely lizards, and the predominant invertebrates, namely tenebrionid beetles. Arguably, they are predominant because they have coped best with the lack of water.

## Lessons for the conservation of the animals of shifting-sand deserts

**Figure 8:**
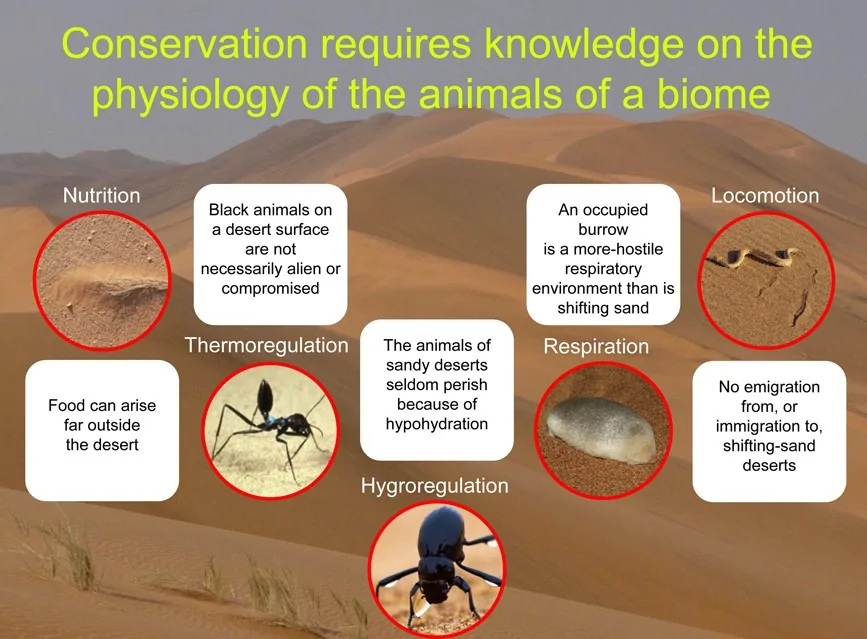
The physiological processes that underpin the welfare of the animals of shifting-sand deserts, with examples of the consequences of special features of those processes for conservation. Photos: background and first inset Shane Maloney, second inset Rüdiger Wehner, third inset Mary Seely, fourth inset Joh Henschel, fifth inset Johan Marais. Copyrights retained by the photographers.

In this review and its companion (Mitchell *et al.,* 2026), we have explored the physiological responses of the animals of shifting-sand deserts to the important abiotic factors that might compromise their function. We have explored how they cope with the mechanics of swimming in sand, how they cope with the heat and cold of desert environments, how they breathe below the sand and how they cope with sparse food and a lack of water. We have established that they cannot leave their fortresses in the sand and that those fortresses are under threat from human abuse of both the shifting-sand deserts themselves and the atmosphere. Figure 8 provides an overview of the mechanisms that we have explored, with an example lesson for conservationists related to each mechanism. What other lessons have we learnt about their physiology that might ameliorate the problems of the conservation of the animals, as the threats impact them?

### For conservation, physiology matters

Conservation practitioners, who, according to Cooke *et al.* (2013, p. 1), set out to ‘identify and mitigate threats, reverse species declines, restore degraded ecosystems, and manage natural resources sustainably’ traditionally have based their decisions predominantly on ecological information, that is, information related to communities and populations. The numerical data largely have been correlations. We do not dispute the value of ecological information, but would argue that it needs to be supplemented with physiological information (Kearney *et al.,* 2013); ‘function’ and ‘ecology’ create an important nexus. ‘Effective conservation and sustainable management of this system {desert} requires that the functional processes which operate are well understood’ (Southgate *et al.,* 1996, p. 273). The main benefit offered by physiology is that it seeks causation, rather than just correlation (Cooke *et al.,* 2013, 2023). Conservation decisions that are derived from physiological information can be based on the mechanisms that animals use to ensure their welfare in whatever circumstances they find themselves. In the following section, we highlight some examples from what we have learnt about the animals of shifting-sand deserts.

An important example concerns the role of water and of food in the survival of animals in shifting-sand deserts. By definition, deserts have available little or no free water, so one might expect that, in times of hardship, some animals would perish from hypohydration, but the physiological evidence is that they seldom do so. As Smith and Morton (1990, p. 268) have said, ‘water limitations have an evolutionary impact on the life histories of arid zone animals, but do not usually limit the survival of individuals directly’. A statistical association between rainfall and survival cannot distinguish between the effect of water scarcity and the effect of food scarcity because the availability of plants in a desert correlates with precipitation. And what does limit the survival of animals in deserts is food (Smith & Morton, 1990; O’Donnell, 2022). Physiological evidence is required, and will be required more in the future as climate change imposes what Wild *et al.* (2025) have called a ‘cost-of-living squeeze’ on the ectotherms of shifting-sand deserts. The ecology and physiology of those animals will be squeezed by climate change because energy expenditure will increase while foraging gains will decrease.

One of the reasons that food availability, rather than water availability, threatens survival is that many desert animals can survive on what they acquire from their food, as preformed water and what they generate as metabolic water. Their water balance is helped because they conserve that water by limiting water loss from the body (see Section ‘The retention of body water in shifting-sand deserts’; Pirtle *et al.,* 2019). For example, the physiological evidence shows that desert lizards pant only *in extremis* and that desert invertebrates typically lose <1% of body mass per day, which is an order of magnitude lower than that of mesic species (Dawson *et al.,* 1989). However physiological investigations reveal subtleties in some of the general patterns too: studies that used doubly labelled water have revealed that the diurnally active gecko *R. bradfieldi* of the Namib Desert cannot survive on preformed water and metabolic water alone; it has to acquire water from elsewhere, with fog being the most-likely source (Murray *et al.,* 2015).

That evidence means that the conservationists of shifting-sand deserts need to be concerned more with food availability than with water availability, for the day-to-day lives of the animals there. Indeed, an increase in water may destroy a desert, as in the case of the Thar Desert (see Mitchell *et al.,* 2026). Physiological studies have revealed that the staple diet in shifting-sand deserts is cellulose (Section ‘Where do the animals of shifting-sand deserts obtain metabolic energy?’), some of which can be derived directly from the sparsely distributed plants that persist in some shifting-sand deserts, but most of which is derived from detritus (Thomas, 1979). Detritivores are the keystone animals of shifting-sand deserts (Thomas, 1979; Smith & Morton, 1990). Detritivores control the residual plant biomass on the desert surface (Section ‘Where do the animals of shifting-sand deserts obtain metabolic energy?’) and also become a source of food for the carnivores and omnivores of the desert. There may be a high correlation between plant density and detritus mass in deserts, but that correlation does not imply that the important source of detritus in a desert is the plants of that desert. In the Namib Desert, at least, detritus is blown in from highlands that are hundreds of kilometres away. Therefore, conservation plans need to attend not just to the desert itself, but to the potentially distant sources of detritus and to any potential barrier to the influx of detritus from those sources into the desert.

Even though conservationists need to pay more attention to food than to water in shifting-sand deserts, and even though the animals of shifting-sand deserts usually do not succumb to hypohydration, droughts may require special interventions. When conservationists are concerned with the welfare and future of the animals of shifting-sand deserts during droughts, is there an overarching principle underlying physiological hydroregulation that could be applied to allay their concerns or, indeed, that might aggravate their concerns? Is there an adaptation that is required of all animals if they are to prosper when water supply is scarce and unpredictable? The answer seems to be ‘no’. As Nagy (1988) pointed out, evolution has delivered multiple and diverse adaptations. There is no short cut to establish which of those adaptations an individual species, or even an individual animal, might or might not have inherited.

There has been a longstanding debate about the merits of the artificial provision of fresh water in designated conservation areas to animals at risk of hypohydration (see Ward, 2016). The opposition to that provision is strongest when the reason for the provision of water is not the welfare of the animals but the satisfaction of tourists, when animals gather at artificial watering points. That gathering does more than damage local vegetation. For example, the gathering alters predator–prey interactions (Juillard *et al.,* 2025). The opponents insist that the animals have evolved to survive without the artificial provision of water, that the generation of gathering points damages the ecology and that the *status ante quo* should be maintained. However, no animal has evolved to combat anthropogenic global warming and the consequential decline in precipitation, especially in deserts. Anthropogenic climate change has added a new dimension to arguments about the provision of artificial water sources.

Physiological investigations have helped to answer the question of why the animals of shifting-sand deserts bury in the sand. Conjecture, supported by isolated measurements, holds that they bury to escape thermal and dehydration stresses on the surface. ‘A valid generalisation is that below-ground climates maintain higher humidity than surface environments’ (Wu *et al.,* 2025, p. 4). Our measurements refute that argument (Seely & Mitchell, 1987). Those measurements were made in the field, and we caution that physiological investigations on the physiology related to the conservation of desert animals should not be confined to laboratories, however convenient and well-controlled those laboratory investigations may be. For example, two populations of the Australian Sand Monitor *Varanus gouldii* had an identical rate of evaporative water loss in the laboratory, but very different rates in the field (Pirtle *et al.,* 2019).

While physiological investigations have supported conservation efforts with explanation of mechanisms, they have revealed some mysteries too. The net cost of transport in desert sand is much higher than the cost of transport on the surface (see Fig. 4). For marsupial moles, the net cost of transport when they are swimming in sand is much higher than is the cost of running on the surface (Fig. 4), yet the marsupial moles forage almost entirely by swimming in the sand. Why they do so is unknown, but needs to be known if they are to be conserved properly. Is it to avoid predators, for example? When they are foraging at times when biotic productivity in the desert is high, Namib golden moles travel shorter distances on the sand surface (Seymour *et al.,* 1998). Do they do that to avoid predation? Another mystery revealed by physiological investigation is that the desert spiny lizard *Sceloporus magister*, native to the Chihuahuan and Sonoran Deserts, needs to be at a temperature of 37–38°C to defaecate (Cowles & Bogert, 1944). If, in the spirit of Hamilton’s theory of maxithermy (see Mitchell *et al.,* 2026), there were advantages to lizards of having a high temperature, few would have anticipated that an ability to defaecate would be one of those advantages.

Finally, in the context of ‘physiology matters’, the research literature on the animals of shifting-sand deserts has been beset, we now think futilely, by discussions about whether the animals cope by ‘physiological’ or by ‘behavioural’ mechanisms (Dawson *et al.,* 1989; Gilmour *et al.,* 2005). Apparently, if a lizard is faced with a rising body temperature and it pants, then that response is physiological, but if it changes posture, then that response is unphysiological. Behavioural responses are changes in function in response to internal or external cues. We and others (Debaere *et al.,* 2024) consider behavioural responses to be facets of physiology, not alternatives to physiology. As Bartholomew (1964, p. 11) pointed out, behavioural ‘adjustments to the environment can be drastic, rapid, precise, and of exquisite flexibility’. Behavioural adjustments presumably are learned; however, an adjustment to a novel environmental challenge would have to rely on physiological responses other than learned behavioural ones (Dawson *et al.,* 1989).

### Why should we conserve shifting-sand deserts and their animals?

We have conveyed our view that shifting-sand deserts are irreplaceable (Mitchell *et al.,* 2026), but that is not a strong reason to conserve them. There are many phenomena in our natural world which are irreplaceable but do not warrant conservation, like the smallpox virus. There is no simple answer to the question of why shifting-sand deserts specifically should be conserved, except the umbrella answer of the International Union for the Conservation of Nature (IUCN) that all biomes and their diversity should be conserved. Is it worth conserving a habitat of dune sands if ‘no productive revenue can be derived from dune sand through crop pastures, mineral exploitation, human habitability or recreation’ (Tsoar, 1990, p. 147–148)? A requirement to limit human activity in dune fields (see Mitchell *et al.,* 2026) may even put a cap on tourism revenue. Le Houérou (1996, p. 168) added another economic factor, namely that of opportunity cost; ‘most developing countries have not the resources to develop or even manage such conservation projects, because there are many more pressing issues and priorities on their budgets’. However, deserts are not voids in the ecosystem; a deterioration of a desert does not leave the ecosystem unaltered (see Mitchell *et al.,* 2026).

According to Le Houérou (1996), we should be grateful that there are indeed so many shifting-sand deserts today: the protected areas of Africa and Asia that were set up in the 1950s and 1960s probably would not be set up today because the pressure on land is so much higher now. The historical evidence, though, is that deserts either are deemed not worthy of protection or that they are so extensive that they do not warrant concern. Only 6% of the world’s deserts and xeric shrublands are under protection compared to 17% of montane grasslands and shrublands (Zhang *et al.,* 2023). We are aware of only one desert sand sea that has UNESCO World Heritage status, the Namib Sand Sea (Seely, 2012). The Dasht-e Lut of Iran (Maghsoudi *et al.,* 2019) also is a World Heritage Site but is mainly a rocky desert, and the Air and Tenere Natural Reserves in northern Niger and the Tassili N’Ajjer National Park in southern Algeria are local areas of the shifting-sand Sahara Desert that are World Heritage Sites.

If we accept the philosophy of the IUCN, then there is a strong case for the conservation of desert dune fields because they are home to many endemic species (Crawford, 1991), and arid zones are particularly vulnerable to the consequences of climate change (Zhang *et al.,* 2023). All dune fields would need to be conserved because there is no ‘representative’ dune field. The lions of the Kruger National Park (South Africa) are the same species as those of the Serengeti National Park (Tanzania), 3000 km away, so the loss of lions from the Kruger National Park might be catastrophic for tourism but would not be catastrophic for conservation. However, the sidewinder of the Namib Desert *Bitis peringueyi* is not the same species, nor even the same genus, as the sidewinder of the Sahara Desert *Cerastes cerastes*; each has to be conserved, separately. The sand seas are fortresses from which resident animals tend not to venture. The marsupial moles of Australian deserts, the fog-basking tenebrionids of the Namib Desert and the silver ants of the Sahara Desert all have unique physiology that is not found anywhere else in the world. In our view, it would be hard to find an argument against their conservation.

## Supplementary Material

Web_Material_coag058

## Data Availability

No data were generated or analysed during this work.
