# Supplementary material for: Physiology and the future of animals in shifting-sand deserts: respiration, energetics and water balance
Source: Conserv Physiol. 2026 Aug 20;14(1):coag058. doi: 10.1093/conphys/coag058 (PMC13491553; doi:10.1093/conphys/coag058)
Supplement: Supplementary file 1 [file web_material_coag058.zip › Mitchell et al. coag058 supplementary material.docx]

**Part 2 supplementary material**

Appendix 1: The mechanics of breathing in sand

When they are on the surface, reptiles conventionally ventilate by lateral compression of the thorax, but in loose sand, granules would fall into the space so created, and prevent re-expansion of the thorax (Arnold, 1995). Sand-swimming lizards and snakes seem to overcome the mechanical forces that oppose breathing by using vertical respiratory movements, an action that also creates on open air space under the thorax and abdomen (Pough, 1969; Etheridge, 2000). Lizards of the genus *Uma*, that engage in quiescent burial, use their forelegs to prevent sand from entering the cavity under the body (Pough, 1969). Both the sandfish *Scincus scincus* and the sympatric sand-swimming skink *Chalcides* (formerly *Sphenops) sepsoides* have concave ventral surfaces when they are buried in the sand (Mosauer, 1932). So too do the quiescent burial snakes *Sonora cincta* (formerly *Chilomeniscus cinctus*) and *S. occipitalis* (formerly *Chionactis occipitalis*) of the shifting-sand deserts of the western USA (Pough, 1969). Though it behaves as a quiescent burier, *S. occipitalis* has the morphology of a sand swimmer, e.g. a small wedge-shaped head continuous with the body (Mosauer, 1935). The Indian sand boa *Eryx johnii*, native to shifting-sand tracts in the Indian subcontinent and adjacent Middle East, switches partially from breathing by lateral compression on the surface to vertical breathing with a concave body when it buries (Pough, 1969). Russell’s sand boa *Eryx conicus,* also native to loose sand in the Indian subcontinent and up to 1 m long, continues to breathe by lateral compression when it buries, but confines itself to a depth of 3-4 mm of sand (Pough, 1969).

As for other arid-zone lizards, the sandfish has a low need for oxygen for its mass, so it is no surprise that its lungs, and its tidal volume, are small (Vihar *et al.,* 2015). Simulations showed that it can inspire and expire into the cavity under its body, and that the air there is appropriate to breathe because enough oxygen can diffuse into it, while enough carbon dioxide can diffuse out of it, through the loose sand (Vihar *et al.,* 2015). The Namib golden mole has a resting metabolic rate of just 22% of that expected for an insectivore of its mass, with the low rate achieved partly because it does not elevate its body temperature above sand temperature (Fielden *et al.,* 1990). Can its respiratory demands in the sand be met without unique physiology? Gasc and colleagues (1986) had reported that its excavation of sand under the head created an air-filled hollow that was maintained during the propulsion phase of sand-swimming. When they watched moles in a glass terrarium, Seymour and Seely (1996) could see no such hollow. Rather, they thought, ventilation occurred via the air spaces of the fur; they estimated that tidal volume was 10% of that space. In the laboratory, they placed six golden moles in a perforated metal cage that was buried 150 mm below the surface of Namib Desert sand in a bucket, for the daylight hours when free-living moles would be below the surface. Through pipes into the cage, they then sampled gas from near the nose of the moles. The lowest partial pressure that they obtained was 18.6 kPa, about 1 kPa lower than that of the free atmosphere, and equal to the oxygen partial pressure at an altitude of about 1000 m. So, the respiratory environment of the moles under 150 mm of Namib sand remained benign, even in the face of the oxygen demand of their mammalian metabolic rate. Even with a mass-specific metabolic rate as low as that of the Namib golden mole, modelling shows that the demands for respiratory gas of animals that swim in Namib sand could not be met if the animals had a mass of more than about 200 g (Seymour and Seely, 1996). The mean mass of the golden moles studied was 19 g; the largest *Gerrhosaurus skoogi* lizard that we weighed had a mass of 120 g (Mitchell *et al.,* 1987).

References

Arnold EN (1995) Identifying the effects of history on adaptation: origins of different sand‐diving techniques in lizards. *J Zool.* 235:351-388.

Etheridge R (2000) A review of lizards of the *Liolaemus wiegmannii* group (Squamata, Iguania, Tropiduridae), and a history of morphological change in the sand-dwelling species. *Herpetol Monogr.* 14:293-352.

Fielden LJ, Waggoner JP, Perrin MR, Hickman GC (1990) Thermoregulation in the Namib Desert golden mole, *Eremitalpa granti namibensis* (Chrysochloridae). *J Arid Environ.* 18:221-237.

Gasc JP, Jouffroy FK, Renous S, Blottnitz FV (1986) Morphofunctional study of the digging system of the Namib Desert golden mole (*Eremitalpa granti* *namibensis*): cinefluorographical and anatomical analysis. *J Zool.* 208:9-35.

Mitchell D. Seely MK, Roberts CS, Pieruszka RD, McClain E, Griffin M, Yeaton R (1987). On the biology of the lizard *Angolosaurus skoogi* in the Namib Desert. *Madoqua* 1987:201-216.

Mosauer W (1932) Adaptive convergence in the sand reptiles of the Sahara and of California: a study in structure and behavior. *Copeia* 1932:72-78.

Mosauer W (1935) The reptiles of a sand dune area and its surroundings in the Colorado Desert, California: a study in habitat preference. *Ecology* 16:pp.13-27.

Pough FH (1969) The morphology of undersand respiration in reptiles. *Herpetologica* 25:216-223.

Seymour RS, Seely MK (1996) The respiratory environment of the Namib Desert golden mole. *J Arid Environ.* 32:453-461.

Vihar B, Wolf C, Böhme W, Fiedler F, Baumgartner W (2015) Respiratory physiology of the sandfish (Squamata: Scincidae: *Scincus scincus*) with special reference to subharenal breathing. *Salamandra* 51:326-334.

.....................................................................................................................................

Appendix 2: How the animals of shifting-sand deserts derive energy from cellulose

Cellulose is a complex polysaccharide that forms the cell wall in plants It consists of glucose molecules linked by β_1,4_ glycosidic bonds, in contrast to the α linkages in carbohydrates that can be digested by most mammals. The digestion of cellulose involves three families of cellulase enzymes that work in series: C1-enzymes that randomly cleave cellulose chains, Cx-enzymes that hydrolyse the resulting short saccharide chains to form cellobiose (disaccharide), and cellobiases (β-glucosidases) that break down cellobiose to monomeric D-glucose (Martin and Martin, 1978; Taylor, 1982; Crawford, 1988). If any one of those steps is absent, the digestion of cellulose fails (Martin, 1983).

Probably no vertebrate species makes its own cellulases, and only a few invertebrate species do so (Martin, 1991). In at least nine orders of insects (Pothula *et al.,* 2019), including desert species, there are species that synthesize cellulases but none, as far as we know, relies entirely on its own cellulases to digest cellulose. Vertebrates and most invertebrates rely on symbionts that synthesize and use cellulases: gastrointestinal bacteria, fungi, protozoans, and other microbial organisms. The hosts derive their energy from the diet by digesting those microorganisms or products that are released by the microorganisms, such as short chain fatty acids in ruminants.

Microorganisms that digest cellulose do not have to be symbionts; self-standing microorganisms can digest cellulose in the soil. However, they require a humid microenvironment that rarely exists in the soil in a shifting-sand desert, but a stable wet environment does occur in the gastrointestinal tracts of invertebrates (Seely and Louw, 1980; Hadley and Szarek, 1981; Crawford and Taylor, 1984; Dawson *et al.,* 1989). The activity of cellulase enzymes is affected only weakly by temperature within the biological range, and so it does not matter that the gastrointestinal tract of invertebrates is not thermally stable (Crawford, 1988).

Invertebrate cellulases, when they are made, and the cellulases of the symbionts that the invertebrates host, act in synergy. Some invertebrates synthesize one or more of the classes of enzyme, but rarely make all three (Martin, 1983); the termite *Macrotermes natalensis*, for example, appears to synthesize Cx-enzymes and cellobiase, but not C1-enzymes, for which it relies on symbionts (Martin and Martin, 1978). The silverfish *Ctenolepisma lineatum* can digest cellulose without symbionts so it is likely that it can synthesize its own C1-enzymes, as well the other classes of cellulase (Martin, 1983).

The gastrointestinal tract of adult tenebrionid beetles contains cellulase that is more active than the cellulase of other desert beetles, as well as some other polysaccharidases (Marcuzzi and Lafisca, 1977). Whether the beetles synthesize their own cellulase, or whether it derives only from resident symbionts, has not been explored, as far as we know.

References

Crawford CS (1988) Nutrition and habitat selection in desert detritivores. *J Arid Environ.* 14:111-121.

Crawford CS, Taylor EC (1984) Decomposition in arid environments: role of the detritivore gut. *S Afr J Sci.* 80:170-176.

Dawson WR, Pinshow B, Bartholomew GA, Seely MK, Shkolnik A, Shoemaker VH, Teeri JA 1989. What's special about the physiological ecology of desert organisms? *J Arid Environ.* 17:131-143.

Hadley NF, Szarek SR (1981) Productivity of desert ecosystems. *BioScience* 31:747-753.

Marcuzzi G, Lafisca MT (1977) The digestive enzymes of some psammophilous tenebrionid beetles from South West Africa. *Madoqua* 1977:191-193.

Martin MM (1983) Cellulose digestion in insects. *Comp Biochem Physiol A Mol Integr Physiol.* 75:313-324.

Martin MM (1991) The evolution of cellulose digestion in insects. *Philos Trans R Soc Lond B Biol Sci.* 333:281-288.

Martin MM, Martin JS (1978) Cellulose digestion in the midgut of the fungus-growing termite *Macrotermes natalensis*: the role of acquired digestive enzymes. *Science* 199:1453-1455.

Pothula R, Shirley D, Perera OP, Klingeman WE, Oppert C, Abdelgaffar HM, Johnson BR, Jurat-Fuentes JL (2019.) The digestive system in Zygentoma as an insect model for high cellulase activity. *PLOS One* 14:e0212505.

Seely MK, Louw GN (1980) First approximation of the effects of rainfall on the ecology and energetics of a Namib Desert dune ecosystem. *J Arid Environ.* 3:25-54

Taylor E.C (1982) Role of aerobic microbial populations in cellulose digestion by desert millipedes. *Appl Environ Microbiol.* 44:281-291.

................................................................................................................................

Appendix 3: Why is sand-swimming metabolically cheaper than is burrowing?

There are several possible reasons why sand-swimming might be less energetically costly than is burrowing. Some of the seemingly attractive reasons have not been supported by experimentation. For example, sand swimming takes place in dry sand and burrows are made in substrates that vary in moisture content, and it might seem logical that more moisture should make sand or soil stickier, and increase the cost to move it while swimming or burrowing. However, in captivity, the marsupial mole *Notoryctes caurinus* would not swim voluntarily in dry sand but would do so in damp sand (Withers *et al.,* 2000). Elsewhere we commented (Mitchell *et al.,* 2026) on the preference that sand-swimming animals have for round granules of specific size. It seems that burrowing in coarse compact substrate elevates the cost above that of sand-swimming, but the experimental evidence for that assertion that burrowing is difficult in coarse sand is inconsistent. When skinks burrowed in coarse versus fine sand, the costs were the same in the two substrates (Wu *et al.,* 2015).

Sand-swimmers and burrowers have to excavate the substrate in their travel direction (usually forwards, but backwards in the spadefoot toad) but sand-swimmers do not incur the considerable cost that is required to transport the substrate away, often to the surface (Vleck, 1979, 1981). Also, it may be that the animals in shifting-sand deserts have been exposed to stronger and longer selection pressures for morphology that is efficient for movement below the surface to evolve, such as the elongated body forms of sand-swimming reptiles and the smooth fur of sand-swimming moles. An example of such evolution is what occurs during pregnancy in the viviparous legless lizards *Acontias* (formerly *Typhlosaurus*) *spp.* The orientation of the embryos has evolved to be parallel to the spine of the mother, rather than curled as they are in mammals, and so the pregnant lizard maintains an elongated body (Huey *et al.,* 1974). Whenever an animal has an elongated body, like the Australian skinks, animals burrow at lower cost than do dumpy animals of the same mass (Wu *et al.,* 2015). The net cost of transport decreases as speed increases through the substrate (Wu *et al.,* 2015) and it may be that sand swimming generally is faster than is burrowing, providing an energetic selection pressure that favours swimming over burrowing.

References

Huey RB, Pianka ER., Egan ME, Coons LW (1974) Ecological shifts in sympatry: Kalahari fossorial lizards (*Typhlosaurus*). *Ecology* 55:304-316.

Mitchell D, Seely MK, Maloney SK (2026) Physiology and the future of animals in shifting‑sand deserts: Living, moving, and thermoregulating in the sand. *Conserv Physiol.*, in press.

Vleck D (1979) The energy cost of burrowing by the pocket gopher Thomomys bottae. *Physiol Zool.* 52:122-136.

Vleck D (1981) Burrow structure and foraging costs in the fossorial rodent, *Thomomys bottae*.  *Oecologia* 49:391-396.

Withers PC, Thompson GG, and Seymour RS (2000) Metabolic physiology of the north-western marsupial mole, *Notoryctes caurinus* (Marsupialia: Notoryctidae). *Aust J Zool.* 48:241-258.

Wu NC, Alton LA, Clemente CJ, Kearney MR, White CR (2015) Morphology and burrowing energetics of semi-fossorial skinks (*Liopholis* spp.).  *J Exp Biol.* 218:2416-2426.

.......................................................................................................

Appendix 4: How dietary plant material accumulates water in deserts

In the Mojave Desert, even otherwise-dry seeds garner substantial water when they are exposed to high ambient humidity and so provide a valuable source of water to the ants of the desert (Morton and MacMillen, 1982). Detritus in the Namib Desert accumulates water when it is exposed to higher ambient relative humidity (Figure 1). The equilibrium water content at 100% humidity was 67%, but that value no doubt overestimates the likely water content of the detritus that is available to detritivores because it would take more than 500 h to attain. The nychthemeral pattern of temperature and humidity in the desert means that detritus usually would be exposed to high humidity for no more than about 12 hours. The values in the plot show the capacity to absorb water after 5 and 10 hours of exposure, and so will be closer to the actual water content of detritus in the environment; the water content of the detritus then approached 20%.

Figure 1. Water content of detritus in the Namib Desert after exposure to air at 20°C at various ambient relative humidities for 5 h and 10 h. Detritus that was wet to begin lost water in a dry environment much more rapidly than it gained water in a humid environment, so preformed water in and on detritus would be available to animals for a limited period only. Derived from Table 1 of Tschinkel (1973). (Data points have been offset by 0.5% laterally to reduce overlap)

[Supplementary material; no ALT TEXT required.]

Relative humidity can exceed 97% on more than 10 days in most months in the Namib Desert. The relative humidity peaks just before dawn (Mitchell *et al.,* 2020), so the preformed water in detritus would be available mostly to those animals that are active before, or soon after, dawn. Beetles in the Namib are normally active later than that, eat only dry detritus (typically < 1% water) during the day (Seely, 1979, 1983) and so do not avail themselves of the pre-dawn water. Instead, those beetles assimilate water directly from fog, and so are active when fog descends on the desert (see Appendix 5). In Israeli deserts, some nocturnally active tenebrionid beetles do consume hygroscopic plant material that has been wet by dew (Broza, 1979; Cloudsley-Thompson, 2001). Quaintly, larvae of the Californian moth *Tinea occidentella* eat only keratin from fur and feathers in carnivore scats and bird pellets. Fog deposits on the keratin in those scats, and the larvae gain most of their water from that source (Strong and John, 2022).

References

Broza M (1979) Dew, fog and hygroscopic food as a source of water for desert arthropods. *J Arid Environ.* 2:43-49.

Cloudsley-Thompson J (2001) Thermal and water relations of desert beetles. *Naturwissenschaften*  88:447-460.

Mitchell D, Henschel JR, Hetem RS, Wassenaar TD, Strauss WM, Hanrahan SA, Seely MK (2020) Fog and fauna of the Namib Desert: past and future. *Ecosphere* 11:e02996.

Morton SR, MacMillen RE (1982) Seeds as sources of preformed water for desert-dwelling granivores. *J Arid Environ.* 5:61-67.

Seely MK (1979) Ecology of a living desert: twenty years of research in the Namib. *S Afr J Sci.* 75:298-303

Seely MK (1983) Effective Use of the Desert Dune Environment as Illustrated by the Namib Tenebrionids. In: Lebrun HM, de Medts AA, Grégoire-Wibo C, Wauthy G, eds. New Trends in Soil Biology. Proceedings of the 8^th^ International Colloquium of Soil Zoology, Dieu-Brichart, Ottignies-Louvain-la-Neuve, pp. 157-168.

Strong DR, John C (2022) Moisture and distribution of a keratophagous moth,*Tinea occidentella*. *Ecol Entomol.* 47:822-830.

Tschinkel WR (1973) The sorption of water vapor by windborne plant debris in the Namib Desert. *Madoqua* 1973:21-24.

....................................................................................................................................

Appendix 5: Fog harvesters of the Namib Desert

The active fog harvesters of the Namib Desert are the fog-baskers *Onymacris unguicularis* and *O. bicolor*, as well as the beetles known as trench-diggers, of the genus *Lepidochora*. In the parts of the desert where active fog harvesters are prevalent, there usually is depositing fog on at least one day of every month. Fog typically occurs at night and the fog-baskers will emerge from the sand before visible droplets form. Somehow those beetles know that the fog is coming, because if there is no fog, they are never on the surface at night (Seely, 1979a). They move to a site on a sand dune near the dune crest that is most appropriate for fog collection and assume a head-down posture, waiting for the fog. In that position, fog droplets accumulate on the dorsal surfaces of the beetle (Hamilton and Seely, 1976). The fog water trickles to their mouthparts, but whether they drink it or whether it enters through the carapace is not yet known (Chakrabarti *et al.,* 2019).

In a single fog event, a fog-basking beetle can consume a mass of water equivalent to one third of its body mass (Hamilton and Seely, 1976). The equivalent would be if a 75 kg human drank 25 litres (100 glasses) of water without interruption. The fog-basking beetles do not drink water that deposits on the dune grass, even though the grass is a very efficient fog harvester (Ebner *et al.,* 2025). They seem to have evolved to harvest fog on vegetationless dunes. Other tenebrionid beetles (Henschel *et al.,* 2001), and the desert scorpion *Parabuthus* *villosus* (Polis and Seely, 1990), do drink water that deposits on the dune grass.

The trench-digging beetles of the genus *Lepidochora* do not collect fog on their bodies as the baskers do, but bulldoze trenches on the dune surface when fog is imminent (Seely and Hamilton, 1976). As the wind blows water-laden fog over the trenches, moisture accumulates on the ridges at a concentration up to six times higher than that of the surrounding sand. The beetles do not dig trenches in the absence of fog. The beetles then can extract some of that moisture, even if there are no visible water droplets. How they do so is not known (Seely, 1979a), but the beetles do not consume the wet sand, as was proposed by Wharton and Richards (1978). “Only water, not sand, was present in amounts sufficient to account for the weight increases measured during the fog” (Seely, 1979 [P. 220]). The capacity to extract water from wet sand is not unique to the beetles. The California legless lizard *Anniella pulchra*, which is found on the sand dunes of the Pacific coast of North America, also can extract water from moist sand in the absence of visible droplets (Fusari, 1985). Its mouthparts must be in contact with the sand, but no sand appears in the gastrointestinal tract.

References

Chakrabarti U, Paoli R, Chatterjee S, Megaridis CM (2019) Importance of body stance in fog droplet collection by the Namib desert beetle. *Biomimetics* 4:59.

Ebner M, Roth-Nebelsick A, Bocherens H, Gschwender F, Baumeister M, Ebner NM, Hohberg K, Gan HY, Schneider C, Maggs-Kölling G *et al.,* (2025) The fog harvesting Namib Desert dune grass *Stipagrostis sabulicola* promotes niche building by modifying substrate and atmosphere conditions. *J Arid Environ.* 227:p.105312.

Fusari MH (1985) Drinking of soil water by the California Legless Lizard, *Anniella* *pulchra*. *Copeia* 1985:981-986.

Hamilton III WJ, Seely MK (1976) Fog basking by the Namib Desert beetle, *Onymacris unguicularis*. *Nature* 262:284-285.

Polis GA, Seely MK (1990) Imbibition of precipitated fog by Namib desert scorpions. *J Arachnol.* 18:362 -363.

Seely MK (1979) Irregular fog as a water source for desert dune beetles. *Oecologia* 42:213-227.

Seely MK, Hamilton III WJ (1976) Fog catchment sand trenches constructed by tenebrionid beetles, *Lepidochora*, from the Namib Desert. *Science* 193:484-486.

Wharton GW, Richards AG (1978) Water vapor exchange kinetics in insects and acarines. *Annu Rev Entomol.* 23:309-328.

.................................................................................................................................

Appendix 6: How animals obtain liquid water from vapour in unsaturated air

The ability to capture water vapour from the air is not ubiquitous amongst insects, nor is it confined to desert insects (Chown *et al.,* 2011). Wigglesworth (1946) thought that the water vapour was absorbed through the carapace. In some invertebrates it may indeed be absorbed through the carapace (e.g., Lees, 1946), but for the few desert insects where the site of water capture is known, it is the gastrointestinal tract. It may be at the mouth end of the tract, as it is in the desert cockroach *Arenivaga investigata* (Edney, 1966; O’Donnell, 1977), which is resident in the sand dunes of the Colorado Desert, or it may be at the rectal end. Many insects (but not *zygentoma*, Machin *et al.,* 1982) have a unique anatomical structure in the rectum that consists of the distal portions of six Malpighian tubules, called the cryptonephridial complex (Beaven and Denholm, 2024). The complex probably evolved to recover water from the excreta (Ramsay, 1964), but in some species, ambient air can be delivered to it by rectal pumping (Coutchié and Machin, 1984), and the complex can extract water vapour from that air and add it to the body water pool.

The mechanism(s) of absorption that different species use to extract water vapour have been under exploration for many years, and still are under exploration (Machin *et al.,* 1982; O’Donnell, 2022). A crucial test in the exploration of mechanisms is to determine whether the rate of extraction depends on a gradient of water vapour pressure, favouring osmotic extraction, or on a gradient of relative humidity, favouring hygroscopic extraction. That test has been applied to the extraction of water vapour from air by the larvae of tenebrionid beetles from the Namib Desert. The test revealed that the extraction was hygroscopic, because the rate of water extraction depended on the gradient in relative humidity, not on a gradient of vapour pressure (Coutchié and Crowe, 1979). Despite these crucial test results, others insist that the capture of water vapour always is osmotic (Larsen *et al.,* 2014). It takes remarkably little metabolic energy to transfer the water from the absorbing surface, whether hygroscopic or osmotic, into body fluids (Larsen *et al.,* 2014; O’Donnell, 2022).

Consistent with the extraction being hygroscopic and not osmotic, though, there is a critical equilibrium relative humidity of ambient air above which water will move to an animal, and below which it will move away from an animal (O’Donnell and Machin, 1988). What that critical equilibrium humidity is depends on the nature of the hygroscopic surface that the animal uses (Winston and Bates, 1960). The critical equilibrium humidity can be extraordinarily low; it is 47.5% for the Namib Desert zygentoman *Ctenolepisma terebrans* after dehydration (Edney, 1971). More typically, the critical equilibrium relative humidity is 80 – 90% (Machin, 1979). That means that those invertebrates of shifting-sand deserts that are capable of extracting liquid water from water vapour in unsaturated air will be able to do so whenever the relative humidity exceeds 80 – 90%. That level of relative humidity occurs during fog, or after dew, and also on those days when the air is nearly saturated. The animals will need to be on the surface or in the upper layers of the sand. Desert rain can increase the water content of sand down to a metre (Southgate *et al.,* 1996; Żmudzka *et al.,* 2014) but depositing fog typically increases the water content of sand down to only 30 mm (Soderberg, 2010). The lives of small desert invertebrates probably depend on the water vapour in that top 30 mm (Mitchell *et al.,* 2020). Some insect species have multiple sensory devices that enable them to seek out the water in those upper layers of sand (Benoit *et al.,* 2023).

References

Beaven R, Denholm B (2025) The cryptonephridial/rectal complex: an evolutionary adaptation for water and ion conservation. *Biol Rev.*100:647-671.

Benoit JB, McCluney KE, DeGennaro MJ, Dow JA (2023) Dehydration dynamics in terrestrial arthropods: from water sensing to trophic interactions. *Annu Rev Entomol.* 68:129-149.

Chown SL, Sørensen JG,Terblanche JS (2011) Water loss in insects: an environmental change perspective. *J Insect Physiol.* 57:1070-1084.

Coutchié PA, Crowe JH (1979) Transport of water vapor by tenebrionid beetles. I. Kinetics. *Physiol Zool.* 52:67-87.

Coutchié PA, Machin J (1984) Allometry of water vapor absorption in two species of tenebrionid beetle larvae. *Am J Physiol - Regul Integr Comp Physiol.* 247:R230-R236.

Edney EB (1966) Absorption of water vapour from unsaturated air by *Arenivaga* sp.(Polyphagidae, Dictyoptera). *Comp Biochem Physiol.*19:387-408.

Edney EB (1971) The body temperature of tenebrionid beetles in the Namib Desert of southern Africa. *J Exp Biol.* 55:253-272.

Larsen EH, Deaton LE, Onken H, O'Donnell M, Grosell M, Dantzler WH, Weihrauch, D (2014.) Osmoregulation and excretion. *Compr Physiol.* 4:405-573

Lees AD (1946) The water balance in *Ixodes ricinus* L. and certain other species of ticks. *Parasitology*  37:1-20.

Machin J (1979) Compartmental osmotic pressures in the rectal complex of *Tenebrio* larvae: evidence for a single tubular pumping site. *J Exp Biol.* 82:123-137.

Machin J, O'Donnell MJ, Coutchié PA (1982) Mechanisms of water vapor absorption in insects. *J Exp Zool.* 222:309-320.

Mitchell D, Henschel JR, Hetem RS, Wassenaar TD, Strauss WM, Hanrahan SA, Seely MK (2020) Fog and fauna of the Namib Desert: past and future. *Ecosphere* 11:e02996.

O’Donnell MJ (1977) Site of water vapor absorption in the desert cockroach, *Arenivaga investigata*. *Proc Natl Acad Sci U S A* 74:1757-1760.

O’Donnell MJ (2022) A perspective on insect water balance. *J Exp Biol.* 225:jeb242358.

O’Donnell MJ, Machin J (1988) Water Vapor Absorption by Terrestrial Organisms. in Wright SH, ed. Advances in Comparative and Environmental Physiology. Volume 2, Springer, Berlin, pp.47–90.

Ramsay JA (1964) The rectal complex of the mealworm *Tenebrio molitor*, L.(Coleoptera, Tenebrionidae). *Philos Trans R Soc Lond B Biol Sci.* 248:279-314.

Soderberg KS (2010) *The role of fog in the ecohydrology and biogeochemistry of the Namib Desert*. Dissertation. University of Virginia, Charlottesville.

Southgate RI, Masters P, Seely MK (1996) Precipitation and biomass changes in the Namib Desert dune ecosystem. *J Arid Environ.* 33:267-280.

Wigglesworth VB (1946.) Water relations of insects. *Experientia* 2:210-214.

Winston PW, Bates DH (1960) Saturated solutions for the control of humidity in biological research. *Ecology* 41:232-237.

Żmudzka E, Woronko D, Dłużewski M (2014) The sources of moisture in the sand dunes–the example of the Western Sahara dune field. *Quaest Geogr.* 33:199-204.

.................................................................................................................................

Appendix 7. The wax waterproofing on the tenebrionids of shifting-sand deserts

The thin wax layer that waterproofs the carapace of beetles is vulnerable to abrasion by sand (e.g., Johnson *et al.,* 2011) but it is renewable (Seely, 1983). The waterproofing also is vulnerable to hyperthermia of the insect surface, because the wax loses its barrier function at a critical temperature that is within the range that the insects of shifting-sand deserts can experience (Larsen *et al.,* 2014). Wigglesworth considered the critical temperature to be 5 – 10°C below the melting point of the wax. There are transitions in the lipid layer at temperatures below that critical temperature that also probably affect permeability (Toolson *et al.,* 1979).

Wigglesworth (1946) thought that the wax was of the same general type as beeswax, but we now have a better understanding of its chemistry. It can contain more than 100 different lipids (O’Donnell, 2022) but the functional barrier to water movement depends primarily on hydrocarbons that have 20 to 40 carbon atoms (Hadley and Louw, 1980). There were 23 different hydrocarbons in the wax on the Namib tenebrionid beetle *Lepidochora discoidalis*, the nocturnal trench-digger, and 33 hydrocarbons in that of the diurnal Namib tenebrionid *Onymacris plana*. At 40°C, the permeability of the cuticle of *O. plana* was about half of that of *L. discoidalis* (Hadley and Louw, 1980). Wigglesworth (1946) reported a critical temperature as low as 31°C for the cockroach *Blattella* (the species was not specified but was presumably *Blattella* *germanica*, the ubiquitous cockroach of the restaurants of Europe). The critical temperature can be as high as 65°C in some species of desert scorpion (Hadley, 1974).

In the tenebrionid beetles of the Sonoran Desert (Hadley, 1979) and the Namib Desert (McClain *et al.,* 1985), and probably of other deserts, the intrinsic layer of wax on the cuticle can be supplemented by a transient wax bloom of diverse colours that is made predominantly of hydrocarbons of the same length as that of the basement wax. The drier the desert environment, the more species of tenebrionid beetle there are that display the bloom (McClain *et al.,* 1985). Several functions have been claimed for the bloom (Hadley, 1984 and see Mitchell *et al.,* 2026), one of which is to decrease the permeability of the cuticle to water even further (McClain *et al.,* 1984, 1985). In the same Namib Desert habitat, the tenebrionid *Zophosis mniszechi*, with a pink wax bloom, had a longer period of surface activity than did its congeneric compatriot *Z. moralesi*, which does not develop a bloom (McClain *et al.,* 1991).

References

Hadley NF (1974) Adaptational biology of desert scorpions. *J Arachnol.* 2:11-23.

Hadley NF (1979) Wax secretion and color phases of the desert tenebrionid beetle *Cryptoglossa verrucosa* (LeConte). *Science* 203:367-369.

Hadley NF (1984) Cuticle: Ecological Significance. In: Bereiter-Hahn J, Matoltsy AG, Richards KS, eds. Biology of the integument. I. Invertebrates. Springer-Verlag, Berlin, pp. 685-693.

Hadley NF, Louw G (1980). Cuticular hydrocarbons and evaporative water loss in two tenebrionid beetles from the Namib Desert. *S Afr J Sci.* 76:298-301.

Johnson RA, Kaiser A, Quinlan M, Sharp W (2011) Effect of cuticular abrasion and recovery on water loss rates in queens of the desert harvester ant *Messor pergandei*. *J Exp Biol.* 214:3495-3506.

Larsen EH, Deaton LE, Onken H, O'Donnell M, Grosell M, Dantzler WH, Weihrauch D (2014) Osmoregulation and excretion. *Compr Physiol.* 4:405-573.

McClain E, Praetorius RL, Hanrahan SA, Seely MK (1984) Dynamics of the wax bloom of a seasonal Namib Desert tenebrionid, *Cauricara phalangium* (Coleoptera: Adesmiini).  *Oecologia* 63:314-319.

McClain E, Seely MK, Hadley NF, Gray V (1985) Wax blooms in tenebrionid beetles of the Namib Desert: correlations with environment. *Ecology* 66:112-118.

McClain E, Kok CJ, Monard LAG (1991) Reflective wax blooms on black Namib desert beetles enhance day activity. *Naturwissenschaften* 78:40-42.

Mitchell D, Seely MK, Maloney SK (2026) Physiology and the future of animals in shifting‑sand deserts: Living, moving, and thermoregulating in the sand.. *Conserv Physiol.*, in press.

O’Donnell MJ (2022) A perspective on insect water balance. *J Exp Biol.* 225*:*jeb242358.

Seely MK (1983) Effective Use of the Desert Dune Environment as Illustrated by the Namib Tenebrionids. In: Lebrun HM, de Medts AA, Grégoire-Wibo C, Wauthy G eds. New Trends in Soil Biology. Proceedings of the 8^th^ International Colloquium of Soil Zoology, Dieu-Brichart, Ottignies-Louvain-la- Neuve, pp.157-168.

Toolson EC, White TR, Glaunsinger WS (1979) Electron paramagnetic resonance spectroscopy of spin-labelled cuticle of *Centruroides sculpturatus* (Scorpiones: Buthidae): correlation with thermal effects on cuticular permeability. *J Insect Physiol.* 25:271-275.

Wigglesworth VB (1946) Water relations of insects. *Experientia* 2:210-214.

.....................................................................................................................................

Appendix 8: How desert animals excrete the products of nitrogen metabolism

The predominant end product of nitrogen metabolism in mammals is urea, which is highly soluble in water (Dantzler and Bradshaw, 2008). At typical mammalian core temperature, the maximum molar concentration of urea in water is about one third (Shnidman and Sunier, 2002), so, when it is in solution, each molecule of urea excreted is accompanied by at least two molecules of water. That maximum concentration decreases to about one quarter at the lower core temperature of the Namib golden mole *Eremitalpa granti namibensis* (Fielden *et al.,* 1990). Thus, the retention of water while the products of nitrogen metabolism are excreted is a formidable physiological problem for the golden mole, and presumably also for the marsupial moles *Notoryctes* sp*.* The problem is compounded because those mammals are insectivorous, and so they have a high-protein diet, and therefore a high rate of nitrogen metabolism, relative to herbivores. Fielden and colleagues (1990 [P. 230-231]) encapsulated the problem: “a physiological factor limiting survival of insectivorous mammals in deserts is the capacity to excrete large quantities of urea in small volumes of urine”. How the marsupial moles have solved the problem is not known. The golden mole has evolved a kidney that can generate a urine concentration of 3820 mosmol.kg^-1^ (Fielden *et al.,* 1990). That is well below the > 9000 mosmol.kg^-1^ that can be attained by the spinifex hopping mouse *Notomys alexis* (Rocha *et al.,* 2021) but is more than four times the urine osmolality that can be achieved by the average human (Buono *et al.,* 2011).

While some birds can produce a urine that is more concentrated than is plasma, reptiles (except some chelonians, Dantzler and Braun, 2008), and insects, cannot do so. They evolved a very different solution to the problem of nitrogen excretion. They eliminate “the poisonous wastes of the vital processes as dry salts of uric acid” (Bailey, 1923 [P. 69]), which are highly insoluble in water. The urate is excreted as a paste (Ward, 2016). Desert scorpions do not excrete urate, but another poorly soluble end product, guanine (Hadley, 1974). The urate can carry excess cations with it too (Dantzler and Braun, 2008).

In some lizards, the ability to retain water is amplified because some of the residual urine that is delivered to the cloaca is diverted retrogradely to the colon, where it can be absorbed (Dantzler and Bradshaw, 2008). Thus, the reptiles and insects, and at least one of the rare mammals (the Namib golden mole), of shifting-sand deserts, have evolved mechanisms that reduce the loss of water when the products of nitrogen metabolism are excreted.

References

Bailey V (1923) Sources of water supply for desert animals. *Sci Mon.* 17:66-86.

Buono MJ, Avila S, Garnero L, Fader L, Kolkhorst FW (2011) The effect of heat acclimation on maximal urine osmolality in humans. *J Therm Biol.*  36:157-159.

Dantzler WH, Bradshaw D (2008). Osmotic and Ionic Regulation in Reptiles. In: Evans DH, ed. Osmotic and Ionic regulation: Cells and Animals. CRC Press, Boca Raton, pp. 443–503.

Dantzler WH, Braun EJ (1980) Comparative nephron function in reptiles, birds, and mammals. *Am J Physiol - Regul Integr Comp Physiol.* 239:R197-R213.

Fielden LJ, Perrin MR, Hickman GC (1990) Water metabolism in the Namib Desert golden mole, *Eremitalpa granti namibensis* (Chrysochloridae). *Comp Biochem Physiol A Mol Integr Physiol.* 96:227-234

Hadley NF (1974) Adaptational biology of desert scorpions. *J Arachnol.* 2:11-23.

Rocha JL, Godinho R, Brito JC, Nielsen R (2021) Life in deserts: the genetic basis of mammalian desert adaptation. *Trends Ecol Evol.*  36:637-650.

Shnidman L, Sunier AA (2002) The solubility of urea in water. *J Phys Chem. 36*:1232-1240.

Ward D (2016) *The Biology of Deserts*. Oxford University Press, Oxford.

...................................................................................................................................

Appendix 9: Respiratory water loss via spiracles

Two main mechanisms are responsible for the discontinuity in exchange of water vapour via the spiracles of insects. The first is the discontinuous gas exchange cycle (DGC) that involves the controlled opening and closing of the spiracles themselves. The second is the opening of spiracles into a sealed subelytral cavity. Ever since it was first discovered, DGC has been assigned a hydroregulatory role, the “hygric hypothesis” (e.g., Cloudsley-Thompson, 2001). But experimental abolition of DGC in the North American ant *Camponotus vicinus* made no difference to its rate of water loss (Lighton and Turner, 2008), a result that is not consistent with a role of DGC in water balance. The evidence that DGC assists with water retention is not strong (Chown *et al.,* 2006; Lighton and Turner, 2008; Contreras *et al.,* 2014). However, a recent meta-analysis of DGC across 17 species from six families in three orders of insect detected a statistically significant hygric effect of DGC (Oladipupo *et al.,* 2022). The desert locust *Schistocerca gregaria* may use the pumping action of DGC to force internal liquid water or water vapour into the haemocoel (Groenewald *et al.,* 2012).

The second mechanism, that involves the opening of abdominal spiracles into the subelytral cavity, occurs in many tenebrionid beetles. The subelytral cavity is a compartment formed by the fusion of the wings over the abdomen and is a feature of several families of beetle. The fusion inevitably renders the beetle flightless (Draney 1993). Spiracles open into the subelytral cavity, though in some beetles respiration can occur through spiracles on the thorax (Chown *et al.,* 2011). Even if present, DGC in subelytral spiracles is not visible in beetles with fused elytra (e.g., Byrne and Duncan, 2003; Schilman *et al.,* 2008); in most but not all tenebrionid beetles, the elytra is fused (Duncan, 2021). The DGC is visible if the elytra is leaky (Duncan, 2021), as it is in some desert insects (Lighton, 1996). Though it serves other purposes, such as expansion of the abdomen (Slobodchikoff and Wismann, 1981), a role in water retention, independent of any role in DGC, has been attributed to the elytra themselves (Draney, 1993). Since the subelytral cavity is saturated with water vapour and isothermal with the abdomen, water does not leave the subelytral spiracles when the cavity is sealed, but the spiracles can take up oxygen from the cavity and release carbon dioxide into it when the partial pressure gradient is favourable. Water and modified air will then be released if the cavity is opened to the air, which it can be actively, via a single small vent called the pygidial cleft. Since the subelytral cavity is opened only occasionally, gas exchange with the outside air occurs in pulses. Thus the same functional outcome of intermittent exchange can be achieved by two very different mechanisms,

References

Byrne MJ, Duncan FD (2003) The role of the subelytral spiracles in respiration in the flightless dung beetle *Circellium bacchus*. *J Exp Biol.* 206:1309-1318.

Chown SL, Gibbs AG, Hetz SK, Klok CJ, Lighton JRB, Marais E (2006) Discontinuous gas exchange in insects: a clarification of hypotheses and approaches. *Physiol Biochem Zool.* 79:333-343.

Chown SL, Sørensen JG,Terblanche JS (2011) Water loss in insects: an environmental change perspective. *J Insect Physiol.* 57:1070-1084.

Cloudsley-Thompson J (2001) Thermal and water relations of desert beetles. *Naturwissenschaften*  88:447-460.

Contreras HL, Heinrich EC, Bradley TJ (2014) Hypotheses regarding the discontinuous gas exchange cycle (DGC) of insects. *Curr Opin Insect Sci.* 4:48-53.

Draney ML (1993) The subelytral cavity of desert tenebrionids. *Fla Entomol.* 76:539-549.

Duncan FD (2021) Respiratory strategies in relation to ecology and behaviour in three diurnal Namib desert tenebrionid beetles. *Insects* 12:1036.

Groenewald B, Hetz SK, Chown SL, Terblanche JS (2012) Respiratory dynamics of discontinuous gas exchange in the tracheal system of the desert locust, *Schistocerca gregaria*. *J Exp Biol.* 215:2301-2307.

Lighton JRB (1996) Discontinuous gas exchange in insects. *Annu Rev Entomol.* 41:309-324.

Lighton JRB, Turner RJ (2008) The hygric hypothesis does not hold water: abolition of discontinuous gas exchange cycles does not affect water loss in the ant *Camponotus vicinus*. *J Exp Biol.* 21:563-567.

Oladipupo SO, Wilson AE, Hu XP, Appel AG (2022) Why do insects close their spiracles? A meta-analytic evaluation of the adaptive hypothesis of discontinuous gas exchange in insects. *Insects* 13:117.

Schilman PE, Kaiser A, Lighton JRB (2008) Breathe softly, beetle: continuous gas exchange, water loss and the role of the subelytral space in the tenebrionid beetle, *Eleodes obscura*. *J Insect Physiol.* 54:192-203.

Slobodchikoff CN, Wismann K (1981) A function of the subelytral chamber of tenebrionid beetles. *J Exp Biol.* 90:109-114.

................................................................................................................................

Appendix 10: How the animals of shifting-sand deserts osmoregulate

When they are dehydrating, some desert tenebrionids defend the concentration of solutes well, while others do not, with no apparent underlying phylogenetic pattern. The tenebrionid *Eleodes hispilabris*, from arid Arizona, USA, regulated haemolymph osmolality well (Riddle *et al.,* 1976), as did larvae of the Namib tenebrionid *Onymacris marginipennis* (Coutchié and Crowe, 1979). The haemolymph osmolality of adults of another Namib tenebrionid *Stenocara gracilipes* increased by only 5% when haemolymph volume decreased by 72% (Naidu, 2001). However, the haemolymph of adults of the Namib tenebrionid *Physadesmia globosa* became hyperosmolar to an extent that would be lethal in vertebrates when haemolymph volume decreased by 61% over ten days of dehydration (Naidu and Hattingh, 1988).

Potentially much more challenging for osmoregulation than is dehydration is rapid rehydration with pure water. To some extent, the problem of osmoregulation during rehydration in insects is ameliorated because osmolytes other than Na^+^, K^+^, and Cl^-^ contribute to the osmolality of their haemolymph, including amino acids, trehalose, and other organic molecules (e.g., Naidu, 1998, 2001; Benoit *et al.,* 2023). As a result, osmolality and electrolyte concentration can be manipulated somewhat independently (Zachariassen *et al.,* 1987). Insects generally are more tolerant of variability in osmolality and electrolyte concentration than are other taxa (Barton-Browne, 1964).

Whatever the physiological mechanisms, most of the animals of shifting-sand deserts seem to manage rapid rehydration successfully (see Table 10 of Mitchell *et al.,* 2020). For example, the osmolality of the haemolymph of *Lepidochora discoidalis* changed hardly at all after the beetle expanded its mass by 41%, with fog water (Hattingh *et al.,* 1984). Some do not do as well; the Namib tenebrionid *Physadesmia globosa*, an opportunistic fog harvester, can become ataxic during rehydration (Naidu and Hattingh, 1988). Some beetles counter the potential dilution of haemolymph during rehydration by sequestering osmolytes during dehydration and then releasing them when the animal rehydrates (e.g., Nicolson, 1980; Naidu and Hattingh, 1986).

In general in lizards, the concentration of plasma electrolytes tends to increase when the lizards are hypohydrated (Bradshaw, 1972). Some urine, which always will be hypo-osmotic with respect to plasma, can be shunted retrogradely into the colon and be absorbed, so attenuating the elevation in plasma electrolyte concentration (Bradshaw, 1975). Though the kidneys of lizards cannot concentrate urine, it is nevertheless the kidneys that are responsible for the regulation of plasma electrolytes. Lizards have a pituitary antidiuretic hormone that is not arginine vasopressin, as it is in mammals, but is arginine vasotocin (Bradshaw, 1975). The plasma sodium concentration is controlled as well in lizards as it is in mammals (Dantzler and Bradshaw, 2008), and they can excrete excess potassium and excess sodium along with the urate pellets that they use to excrete the products of nitrogen metabolism. However, that mechanism cannot be used to excrete chloride or other anions (Clarke and Nicolson, 1994; Hazard *et al.,* 2010), which require another route of excretion.

The nasal glands can be used to excrete excess chloride and other electrolytes, though not all lizards have them (Bradshaw, 1972). Nasal glands may escape attention in the lizards of shifting-sand deserts; salt from the glands tends to be observed more often in lizards in captivity than in the field, because the desert sand scours the salty exudate from the body surface (Clarke and Nicolson, 1994). The salt glands of reptiles, including lizards, excrete a wider spectrum of electrolytes than do those of marine birds (Dantzler and Bradshaw, 2008), though the salt glands of both lizards and marine birds excrete chloride (Hazard *et al.,* 2010). As Dantzler and Bradshaw put it, “little is known of the actual mechanism by which a hyperosmotic salt solution is generated by the reptilian nasal gland” (Dantzler and Bradshaw, 2008 [P.475]). The make-up of electrolytes that are excreted seems to be under the control of the conventional hormones that are responsible for electrolyte management, like aldosterone (Benoit *et al.,* 2023; Bradshaw, 1975). The salt glands of the desert iguana *Dipsosaurus dorsalis* are not activated by sodium overload, however (Hazard, 2001).

References

Barton-Browne LB (1964) Water regulation in insects. *Annu Rev Entomol.*  9:63-82.

Benoit JB, McCluney KE, DeGennaro MJ Dow JA (2023) Dehydration dynamics in terrestrial arthropods: from water sensing to trophic interactions. *Annu Rev Entomol.*  68*:*129-149.

Bradshaw SD (1972) The endocrine control of water and electrolyte metabolism in desert reptiles. *Gen Comp Endocrinol.* 3:360-373.

Bradshaw SD (1975) Osmoregulation and pituitary-adrenal function in desert reptiles. *Gen Comp Endocrinol.* 25:230-248.

Clarke BC, Nicolson SW (1994) Water, energy, and electrolyte balance in captive Namib sand-dune lizards (*Angolosaurus skoogi*). *Copeia* 1994:p962-974.

Coutchié PA, Crowe JH (1979). Transport of water vapor by tenebrionid beetles. II. Regulation of the osmolarity and composition of the hemolymph. *Physiol Zool.* 52:88-100.

Dantzler WH, Bradshaw D (2008). Osmotic and Ionic Regulation in Reptiles. In: Evans DH, ed. Osmotic and Ionic Regulation: Cells and Animals. CRC Press, Boca Raton, pp. 443–503.

Hattingh J, Ganhao M, Naidu SG, (1984) Haemolymph composition in Namib Desert tenebrionid beetles. *Comp Biochem Physiol A Mol Integr Physiol.* 78541-545.

Hazard LC (2001) Ion secretion by salt glands of desert iguanas (*Dipsosaurus dorsalis*). *Physiol Biochem Zool.* 74:22-31.

Hazard LC, Lechuga C, Zilinskis S (2010) Secretion by the nasal salt glands of two insectivorous lizard species is initiated by an ecologically relevant dietary ion, chloride. *J Exp Zool A Ecol Genet Physiol.* 313:442-451.

Mitchell D, Henschel JR, Hetem RS, Wassenaar TD, Strauss WM, Hanrahan SA, Seely MK (2020) Fog and fauna of the Namib Desert: past and future. *Ecosphere* 11:e02996.

Naidu SG (1998) Haemolymph amino acid, sugar and glycerol levels in the Namib Desert tenebrionid *Stips stali* during dehydration and rehydration. *Comp Biochem Physiol A Mol Integr Physiol.* 119:477-484.

Naidu SG (2001) Water balance and osmoregulation in *Stenocara gracilipes*, a wax-blooming tenebrionid beetle from the Namib Desert. *J Insect Physiol.* 47:1429-1440.

Naidu SG, Hattingh J (1986) Water balance and osmoregulation in *Stips stali*, a nocturnal tenebrionid beetle from the Namib Desert. *J Insect Physiol.* 32:891-896.

Naidu SG, Hattingh J (1988) Water balance and osmoregulation in *Physadesmia globosa*, a diurnal tenebrionid beetle from the Namib desert. *J Insect Physiol.* 34:911-917.

Nicolson SW (1980) Water balance and osmoregulation in *Onymacris plana*, a tenebrionid beetle from the Namib Desert. *J Insect Physiol.* 26:315-320.

Riddle WA, Crawford CS, Zeiton AM (1976) Patterns of hemolymph osmoregulation in three desert arthropods. *J Comp Physiol.* 112:295-305.

Zachariassen KE, Kamau JM, Maloiy GM (1987) Water balance and osmotic regulation in the East African tenebrionid beetle *Phrynocolus* *petrosus.* *Comp Biochem Physiol A Mol Integr Physiol.* 86:79-83.

.......................................................................................................................................
